# AreTomoLive: automated reconstruction of comprehensively corrected and denoised cryo-electron tomograms in real time and at high throughput

**DOI:** 10.1038/s41592-026-03093-y

**Published:** 2026-05-25

**Authors:** Ariana Peck, Yue Yu, Mohammadreza Paraan, Dari Kimanius, Utz H. Ermel, Joshua Hutchings, Jonathan Schwartz, Daniel Serwas, Hannah Siems, Norbert S. Hill, Mallak Ali, Julia Peukes, Garrett A. Greenan, Shu-Hsien Sheu, Elizabeth A. Montabana, Bridget Carragher, Clinton S. Potter, David A. Agard, Shawn Zheng

**Affiliations:** 1 Biohub, Redwood City, CA USA; 2 Department of Biochemistry and Biophysics, University of California, San Francisco, San Francisco, CA USA

**Keywords:** Imaging, Cellular imaging, Software, Computational biophysics

## Abstract

High-throughput data processing is necessary to realize the full potential of cryo-electron tomography and subtomogram averaging. The field’s fragmented software landscape remains a considerable hurdle to this end. Here we present AreTomoLive, an automated preprocessing pipeline composed of two GPU-accelerated packages. The first, AreTomo3, streamlines tomographic alignment and reconstruction, with new features to fully account for sample geometry and locally correct the contrast transfer function. The second package, DenoisET, leverages AreTomo3’s comprehensively corrected tomograms to retain more intermediate-resolution features during contrast enhancement. Collectively, this pipeline prioritizes automation to support data preprocessing concurrent with data collection at scale.

## Abstract (web summary)

AreTomoLive is an accelerated preprocessing pipeline for cryo-electron tomography that streamlines tomographic alignment, reconstruction and contrast enhancement. This pipeline prioritizes automation and throughput to deliver comprehensively corrected and denoised tomograms, including during data collection at scale.

## Main

**Extended Data Fig. 1:**
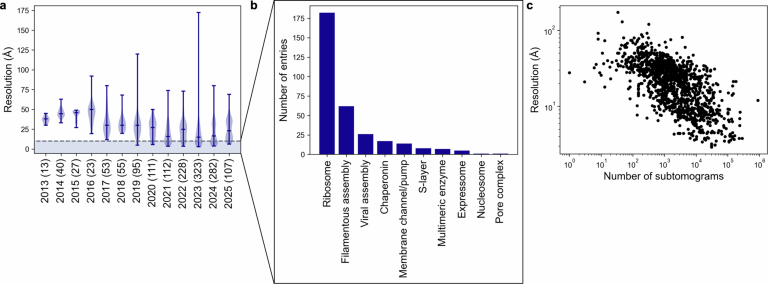
Characteristics of in situ subtomogram averages deposited in the Electron Microscopy Data Bank. Structures determined by STA from cell and tissue samples were included in the analysis. **a**. The resolution of these structures is shown as a function of year, with the dashed line indicating 10 Å and the number of depositions noted in parenthesis. The vertical and horizontal lines respectively correspond to the full resolution range and median resolution of the depositions. **b**. The molecular identities of 10 Å or higher resolution structures are categorized. Ribosomes, which are abundant, and viral and filamentous assemblies, which often have high symmetry, collectively account for 84% of these subnanometer resolution structures. **c**. Each structure’s resolution is plotted as a function of the number of subtomograms used for averaging.

Cryo-electron tomography (cryoET) is a powerful technique for visualizing the molecular machinery that drives cellular function^1^. Subtomogram averaging (STA) enables structure determination from cryoET data in the native cellular environment^2^. However, subnanometer-resolution reconstructions are difficult to achieve for in situ molecules at low abundance^3–6^ (Extended Data Fig. 1). Improvements in throughput, alignment accuracy and contrast are critical to extend high-resolution STA to new molecular species^1^. Higher throughput is needed for STA to overcome the extremely low signal-to-noise ratio (SNR) of cryoET data. Alignment accuracy is essential to recover high-resolution information, which is attenuated by beam-induced sample motion and signal delocalization by the contrast transfer function (CTF)^7,8^. Finally, contrast enhancement is often critical to locate target molecules and interpret their cellular context.

The recent development of parallel acquisition schemes has accelerated data collection^9,10^. Despite this, the software landscape for cryoET data preprocessing—spanning motion correction of the acquired tilt-movies to contrast enhancement of aligned tomograms—remains fragmented. Many pipelines assemble third-party software packages that rely on distinct coordinate conventions, file formats and software dependencies, making them difficult to maintain and increasing I/O overhead^11–13^. To address these barriers, AreTomoLive provides an integrated pipeline through its constituent packages AreTomo3 and DenoisET, which together automate tomographic alignment, reconstruction and contrast enhancement for real-time data preprocessing. AreTomo3 implements new features to improve alignment and reconstruction accuracy, which in turn increases the retention of intermediate-resolution features during contrast enhancement by DenoisET.

## Results

### Real-time tomographic alignment and reconstruction by AreTomo3

**Fig. 1:**
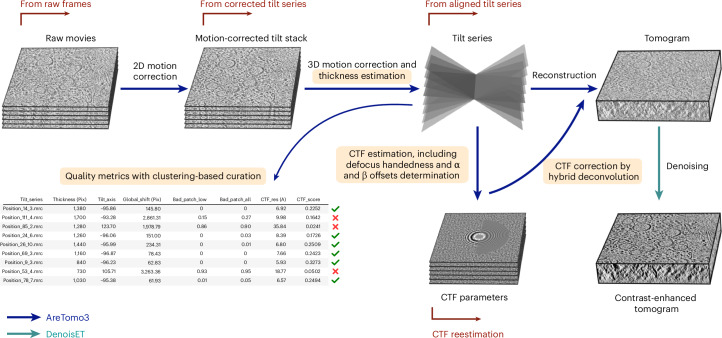
AreTomoLive provides a fully automated pipeline for real-time tomographic alignment, reconstruction and contrast enhancement. AreTomoLive is composed of two GPU-accelerated packages, AreTomo3 and DenoisET. AreTomo3 performs the tasks indicated by the dark-blue arrows to provide comprehensively corrected tomograms minutes after each raw tilt movie is collected. Beyond integrating the MotionCor2 (ref. ^14^) and AreTomo^8^ software packages, AreTomo3 introduces new features (highlighted text) to improve the accuracy of alignment and reconstruction and facilitate data curation. The red arrows indicate the multiple entry points that allow users to efficiently reprocess data from an intermediate state. The second component of AreTomoLive, DenoisET, runs in parallel with AreTomo3 to train and then apply a machine learning-based denoising model to the tomograms that AreTomo3 continually writes to disk. 2D, two-dimensional; 3D, three-dimensional.

**Extended Data Fig. 2:**
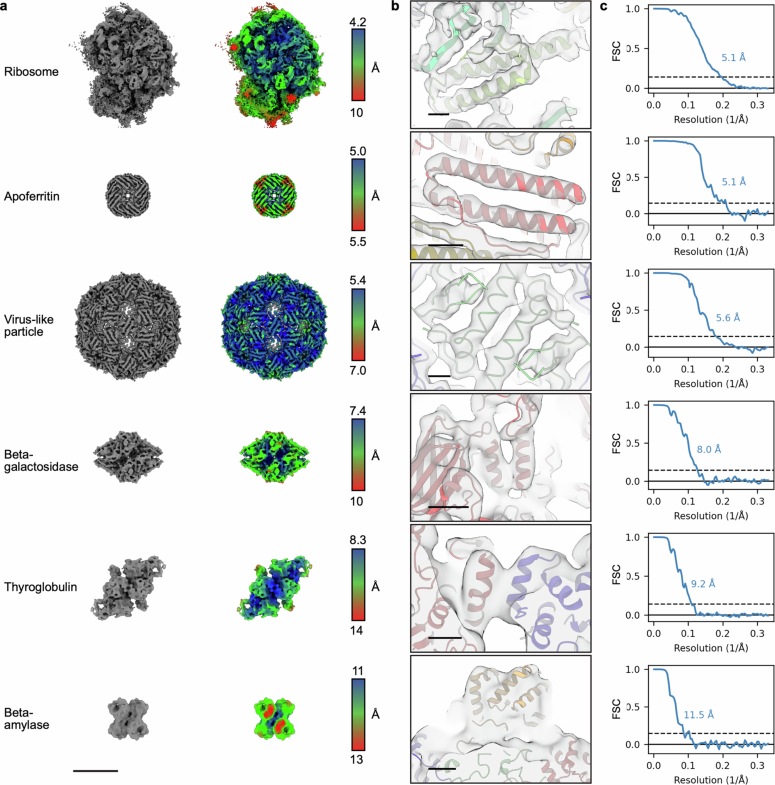
Multiple subnanometer and one near-10 Å resolution reconstructions from data pre^−^processed by AreTomoLive and with STA performed in RELION-5. Particles were picked in tomograms reconstructed by AreTomo3 and contrast-enhanced by DenoisET from a benchmark dataset^17^. These particles were then extracted by RELION-5^16^ from AreTomo3’s motion-corrected tilt-series as 2D particle stacks. Bayesian polishing and CTF refinement were skipped so that the tomographic alignments and CTF estimates were fully derived from AreTomo3 without further refinement in RELION-5. **a**. 3D reconstructions are shown for the indicated particle, with local resolution visualized on the maps on the right. The scale bar indicates 10 nm. **b**. A portion of each reconstruction is displayed, with the density map overlaid on the following fitted reference structure from the Protein Data Bank: ribosome, 6QZP; apoferritin, 7VD8; virus-like particle, 1DWN; beta-galactosidase, 6DRV; thyroglobulin, 7B75; beta-amylase, 1FA2. The scale bar indicates 1 nm. **c**. The Fourier shell correlation (FSC) between half-maps was used to estimate the global resolution. Performing Bayesian polishing and CTF refinement improved the global resolution to 3.6, 3.9, and 4.1 Å for the ribosome, apoferritin, and virus-like particle maps, respectively; these steps were not performed for the other particles due to their lower resolution. The 0.9-1.5 Å improvement in resolution is very similar to the pixel size (1.51 Å), suggesting that AreTomo3’s alignment accuracy approached the resolution limit of the tilt-series data.

The first component of AreTomoLive, AreTomo3, streamlines tomogram reconstruction from raw tilt-movies and is divided into three modules (Fig. 1). The first module inherits corrections for anisotropic beam-induced motion in two dimensions from MotionCor2 (ref. ^14^) and assembles the motion-corrected micrographs into tilt series. The second module performs iterative CTF estimation and tomographic alignment, extending the alignment procedure developed in AreTomo^8^. The third module applies the measured global and local alignments to the tilt series to reconstruct tomograms, with corrections for the local CTF and α_0_ tilt offset optionally applied. In addition to tomograms, AreTomo3 outputs alignment parameters, quality metrics for each tilt series (Supplementary Note 1) and the tilt series and CTF parameters required by modern STA workflows^15,16^ to facilitate data curation and downstream processing. Extended Data Figure 2 demonstrates the usefulness of AreTomo3’s metadata and processing results for STA in RELION-5 (ref. ^16^). AreTomo3’s tomographic alignments and CTF estimates were of sufficiently high quality to reconstruct six molecular species spanning 260 kDa–4.3 MDa from a benchmark dataset^17^ at resolutions of 5.1–11.5 Å, even without CTF refinement or Bayesian polishing. The performance and scaling behavior for individual preprocessing steps are listed in Extended Data Table 1 and compared to other pipelines in Supplementary Note 2.

**Extended Data Fig. 3:**
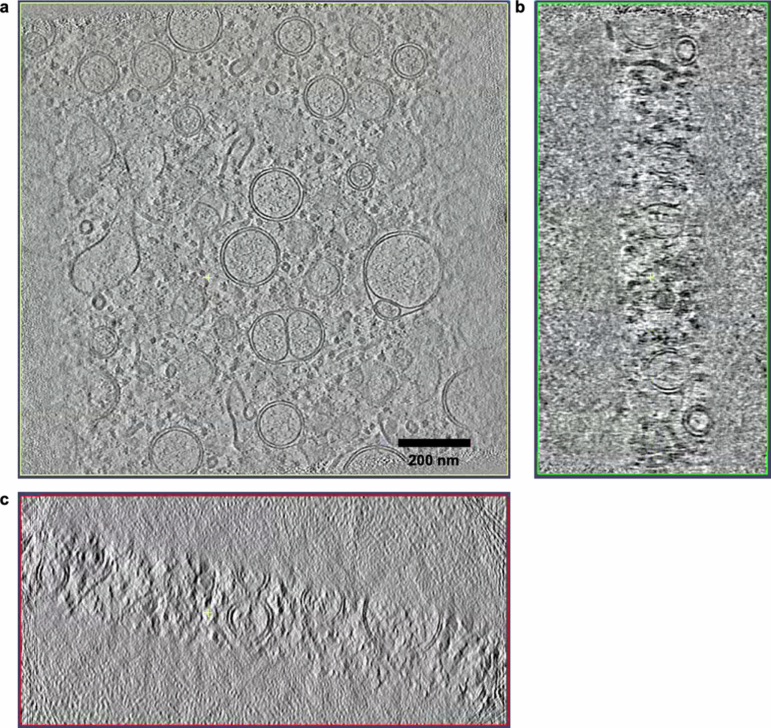
Orthogonal tomographic slices from a tomogram of a FIB-milled lamella of an arterivirus-infected cell. **a**. A slice from the *xy* plane. **b**. A slice from the *yz* plane shows a noticeable β_0_ tilt. The estimate by AreTomo3 is −2.2°. **c**. A slice from the *xz* plane shows the α_0_ tilt. The estimate by AreTomo3 is 12.3°.

**Extended Data Fig. 4:**
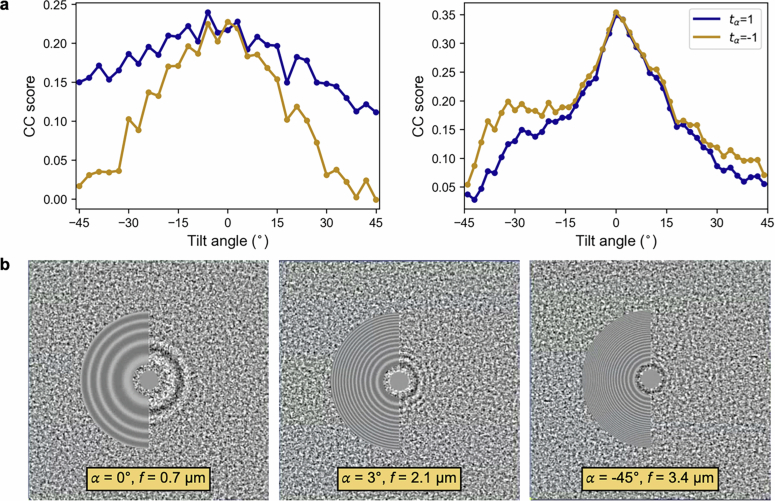
AreTomo3’s CTF refinement scheme resolves the tilt axis directionality and reliably estimates defocus for challenging data acquisition schemes. **a**. AreTomo3 resolves the tilt axis directionality during CTF refinement by comparing CTF scores across the full tilt range for the two values of the tilt axis ambiguity factor (*t*_a_ = ±1). In the example on the right, the tilt axis ambiguity factor was determined to be negative, requiring correction of the initial tilt axis angle by subtracting 180°. **b**. A tilt-series of affinity-captured lysosomes was collected according to a modified TYGRESS^40^ scheme. The 0° image was collected with 10-fold more electron dose and at a significantly lower defocus setting than the tilted images to facilitate template matching. Despite these dose and defocus discontinuities across the tilt-series, AreTomo3 reliably measured the CTF parameters, including at high tilt angles.

Beyond seamlessly integrating MotionCor2 (ref. ^14^) and AreTomo^8^, AreTomo3 implements new features to achieve high alignment and reconstruction accuracy. One such feature performs robust estimation of the CTF parameters. This measurement is especially difficult for tilt series, which are characterized by weak Thon rings that are further attenuated by the oblique defocus gradient across each tilt image (Extended Data Fig. 3). For robust estimation, AreTomo3 jointly optimizes the tilt offsets, tilt axis directionality (Extended Data Fig. 4a) and per-tilt CTF parameters from overlapping tiles, which are spectrally rescaled^18^ based on their local defocus. This full accounting of the sample geometry increases the spatial coherence of the tiles’ power spectra, which in turn improves CTF parameter estimation. To compensate for the reduced signal at higher tilt angles, AreTomo3 refines only the defocus for tilt images beyond ±30° and bootstraps the other parameters to the nearest less-tilted image. This strategy has shown robust CTF determination even at high tilt angles and modified acquisition schemes with large defocus discontinuities across the tilt series (Extended Data Fig. 4b). Comparison of AreTomo3’s defocus estimates with other widely used software packages showed high agreement with WarpTools^19^ and improved accuracy compared to CTFFIND5 (ref. ^20^), especially at high tilt angles (Supplementary Note 3).

**Extended Data Fig. 5:**
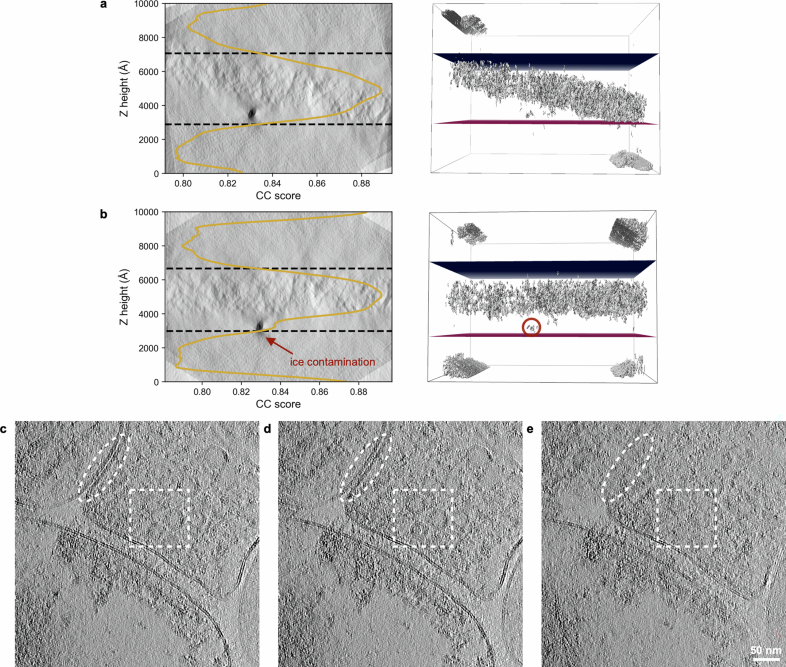
AreTomo3’s runtime measurement of sample thickness. **a**. Slices through the *xz* plane (left) and 3D views (right) are visualized for a tomogram of coronavirus-induced double-membrane vesicles. The cross-correlation (CC) scores between adjacent slices along the *z*-axis are overlaid in yellow and were used to estimate the sample boundaries (dashed lines and colored planes in the left and right panels, respectively). **b**. Correction of the α_0_ tilt offset levels the sample within the tomogram and improves the accuracy of the thickness measurement. However, the presence of a large ice crystal (red circle) artificially inflates the measured thickness. **c-e**. Correctly estimating the sample thickness improves local alignment accuracy. The same tilt-series of purified synaptosomes was aligned with three different values for the sample thickness used during the alignment process: **c**. 770 Å; **d**. 1500 Å, which is the sample thickness estimated by AreTomo3; and **e**. 3800 Å. The membranes labeled inside the oval in **d**. are better defined than in **e**., and the presynaptic vesicles outlined in the square box are most distinct in **d**.

A second innovation in AreTomo3 provides runtime measurements of sample thickness to improve tomographic alignment. Previously in AreTomo^8^, users could specify the thickness parameter used for alignment. However, sample thickness can be difficult to predict before reconstruction, and a single value is often unsuitable even for targets from the same sample. Instead of relying on one user-specified value, AreTomo3 estimates the sample thickness for each tilt series by analyzing the cross-correlation profile along the depth of an intermediate tomogram reconstructed after global alignment. This correlation decays outside the sample due to the absence of structurally continuous features and can thus be used to estimate the sample boundaries. This approach generalizes to diverse samples but can be confounded by ice contamination and assumes that the sample is leveled in the tomogram (Extended Data Fig. 5a,b). Consequently, lamella samples with a large α_0_ tilt offset should be reconstructed with this offset corrected to exclude the vacuum above and below the inclined plane of the sample. Correctly estimating the sample thickness is important for alignment accuracy. AreTomo3 computes the local alignments by performing projection matching between the globally aligned tilt series and a reference tilt series generated from an intermediate tomogram. An overestimate of the sample thickness introduces a vacuum into this intermediate tomogram, while an underestimate restricts the available structural information. Both scenarios yield poor reference tilt series and negatively impact reconstruction quality (Extended Data Fig. 5c).

**Extended Data Fig. 6:**
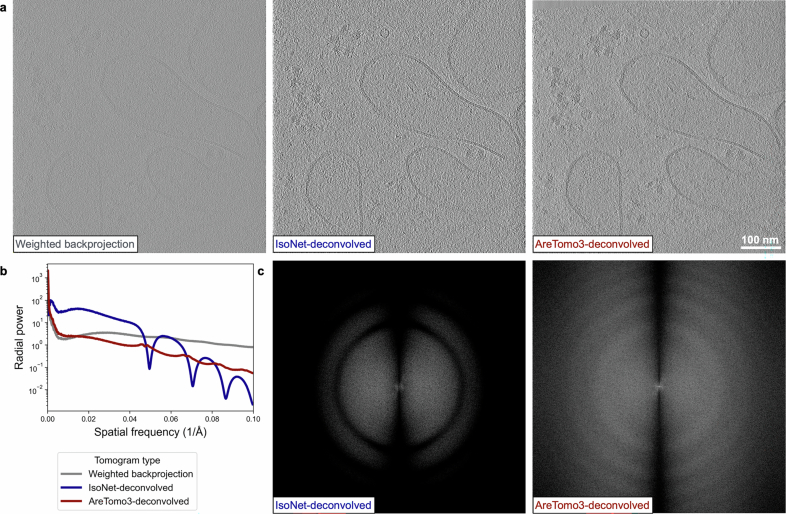
Comparison of CTF deconvolution in AreTomo3 and IsoNet. **a**. A tomogram from a benchmark dataset reconstructed by weighted backprojection (left) was CTF-deconvolved by IsoNet^22^ (center) or AreTomo3 (right). IsoNet applies a Wiener filter-like deconvolution post-reconstruction using a single defocus value for the full volume, whereas AreTomo3 applies a hybrid deconvolution locally to the tilt-series to account for the defocus gradient. **b**. The radial intensity profiles and **c**. slices of the CTF-deconvolved tomograms’ Fourier transforms are compared.

AreTomo3 also implements a new local CTF correction to account for the oblique defocus gradient across each tilted image. This local correction is applied to the tilt series and transitions from Wiener filtering to phase flipping with increasing spatial frequency. This hybrid scheme balances the Wiener filter’s ability to compensate for the CTF-induced amplitude oscillations that dominate at low resolution and its tendency to distort noise statistics at high resolution^21^. Similarly to Warp^19^, AreTomo3’s CTF correction also applies a low-pass filter of adjustable strength. Extended Data Fig. 6 compares AreTomo3’s local CTF correction to the approach implemented in IsoNet^22^, which is based on Warp^19^. In contrast to AreTomo3, IsoNet deconvolves the CTF from tomograms based on a single defocus value rather than locally on the tilt series and applies a Wiener-like filter across all spatial frequencies. As a result, the drop in intensity at the CTF’s zero-crossings is more pronounced in the IsoNet case (Extended Data Fig. 6b,c). Although in principle there should be no information transfer at these spatial frequencies, in practice the intensity will be nonzero due to noise, and sharp features in Fourier space could be detrimental downstream tasks.

### Automated contrast enhancement by DenoisET

**Fig. 2:**
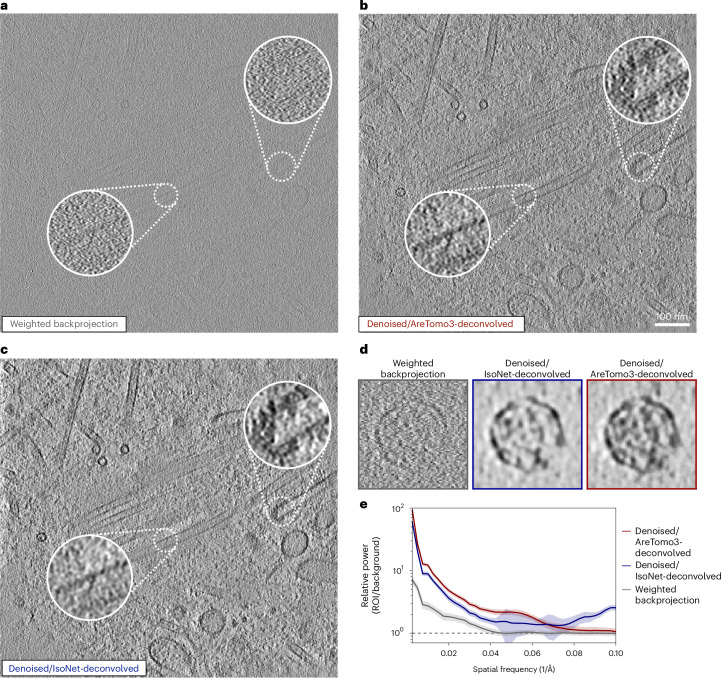
AreTomo3’s local CTF correction improves the retention of intermediate-resolution features during denoising. **a**–**c**, A 10-Å slice from a lamella of a cilium and centrosome is compared from the undenoised tomogram (**a**) and after denoising the AreTomo3-corrected (**b**) versus IsoNet^22^-corrected (**c**) tomograms. The insets highlight proteins embedded in the membrane and arrayed along the filament. **d**, Representative virus-like-particles from a benchmark dataset^17^ are compared from the indicated tomograms. **e**, Contrast enhancement was estimated as the ratio of the average power spectra of these regions of interest (ROIs) to background regions for the indicated tomogram type. These ratios are plotted as dark lines, with the standard deviation indicated by shading.

**Extended Data Fig. 7:**
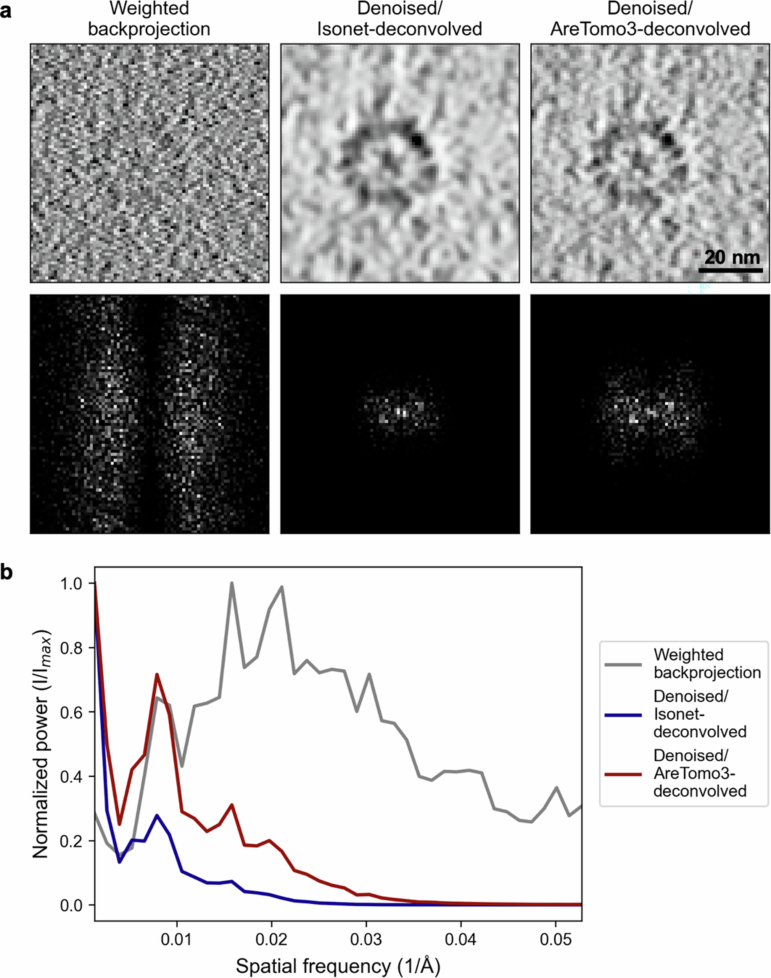
Comparison of the impact of different CTF deconvolution approaches on subsequent denoising. **a**. A cross-section through a microtubule from a lamella of a cilium and centrosome is shown from the undenoised tomogram (left) and the indicated denoised tomogram, where denoising was performed after CTF deconvolution by either IsoNet^22^ (center) or AreTomo3 (right). The lower panel shows the Fourier transform of each cross-section. In real space, individual protofilaments are better resolved in the AreTomo3 case. Correspondingly in Fourier space, features are observed to higher resolution. **b**. The normalized radial intensity profiles of the cross-sections show superior retention of intermediate resolution features in the AreTomo3 case.

Noise2Noise-based contrast enhancement^23^ is integrated into AreTomoLive through its second component, DenoisET. Given that many recent three-dimensional denoising methods for cryoET data train on tomograms that have been CTF-deconvolved following the Warp/IsoNet approach^3,22,24^, AreTomo3’s and IsoNet’s distinct approaches to CTF correction on subsequent denoising were compared. We found that a stronger low-pass filter was needed to achieve the highest quality denoising in the IsoNet case compared to AreTomo3’s default filter strength (Extended Data Figs. 6 and 7). However, applying a strong low-pass filter blurs intermediate-resolution features that ideally the neural network would learn during training and retain during inference. Consistent with this, intermediate-resolution features were better preserved after denoising tomograms that were CTF-corrected by AreTomo3 without introducing high-resolution artifacts as observed in the IsoNet case (Fig. 2 and Extended Data Fig. 7).

**Extended Data Fig. 8:**
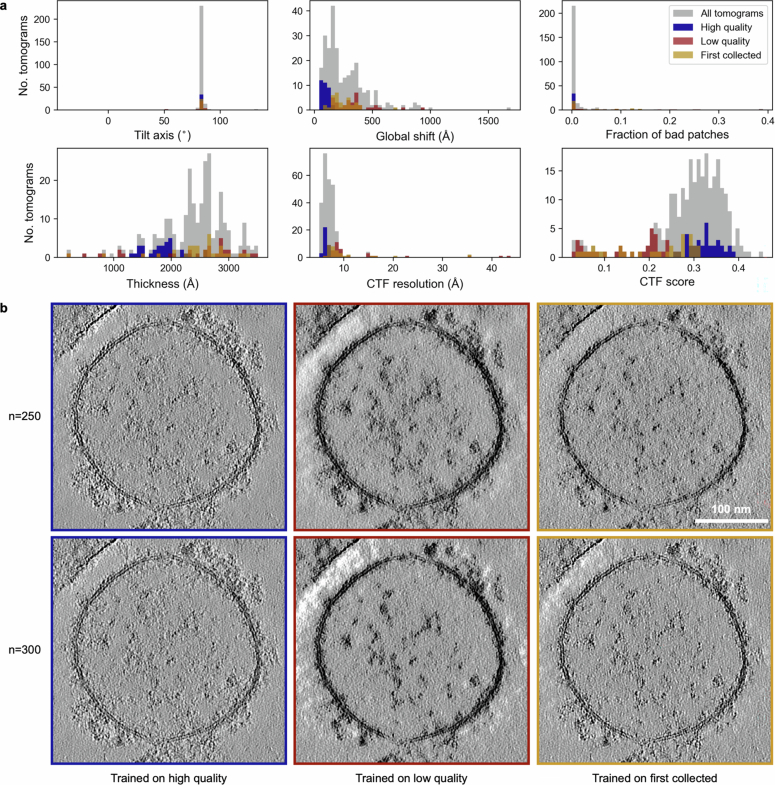
Curating training data using AreTomo3’s quality metrics yields more robust denoising. **a**. Tomograms from a dataset of purified lysosomes were selected if they met certain thresholds for the six metrics shown, resulting in a high-quality training set of 34 tomograms. For comparison, training sets were also generated from 34 tomograms with low CTF scores and poor alignment and the first 34 tomograms collected during this session. The latter mimics the scenario of running denoising live, except without any data curation. **b**. The resulting models from these different training sets were used to denoise a tomogram that was not included in any of the training sets. To examine consistency across hyperparameter space, the quality of denoising was compared by varying the number of subvolumes extracted per tomogram per training epoch (n) during model training.

**Extended Data Fig. 9:**
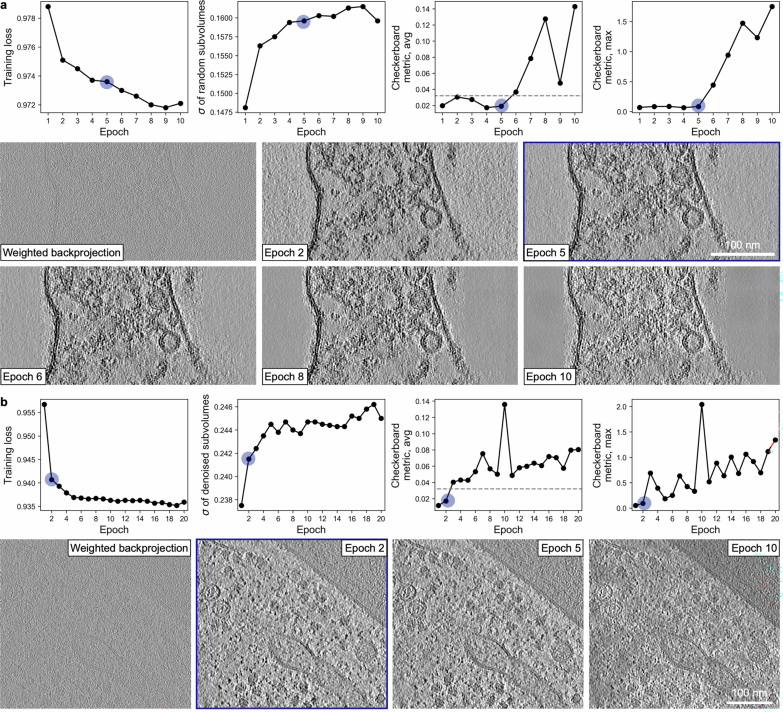
Automated termination of training based on the emergence of checkerboard artifacts. Compared to the training loss and standard deviation of denoised subvolumes, metrics quantifying the appearance of checkerboard artifacts (see Methods) in the denoised tomograms were found to be more sensitive to over-denoising. These metrics are plotted as a function of training epoch (upper), and slices through the indicated tomogram are visualized (lower) for a dataset of **a**. purified synaptosomes and **b**. yeast lamella. In both cases, the circled epoch was chosen based on a maximum threshold of 0.034 for the average checkerboard metric (dashed line).

Compared to prior implementations of the Noise2Noise algorithm for cryoET data^25,26^, DenoisET focuses on providing a fully automated approach. Specifically, tomograms are automatically selected based on AreTomo3’s quality metrics to provide a high-quality training set (Extended Data Fig. 8), and DenoisET initiates training once sufficient high-quality tomograms are detected. Training is automatically terminated based on the emergence of checkerboard artifacts in the denoised tomograms, which was observed to be more sensitive to over-denoising than the loss function (Extended Data Fig. 9). Automating this step is critical since the common practice of selecting the optimal training epoch based on visual inspection is unviable for real-time processing^24,27^. Once training finishes, DenoisET performs inference on all available tomograms and transitions to monitoring for additional tomograms to denoise.

## Discussion

Together, AreTomo3 and DenoisET provide an integrated pipeline for real-time and fully automated cryoET data preprocessing. However, there is still room to better leverage AreTomo3’s comprehensive corrections for downstream tasks. In particular, the RELION-4/5 STA workflows^15,16^ that extract data from tilt series currently incorporate only AreTomo3’s global alignments but neither its local alignments nor its correction of the α_0_ tilt offset. Future work will focus on defining a common deformation model to bridge this gap since locally aligned data are expected to accelerate the convergence of subtomogram polishing. AreTomoLive could also be extended to iteratively refine tomographic alignment. Specifically, the denoised tomograms could be forward-projected into contrast-enhanced tilt series used to optimize the local alignments and tilt angles under a Bayesian framework. The increased alignment accuracy from this subvolume polishing scheme would yield more spatially coherent tomograms, in turn providing the basis for superior denoising. Continued efforts to improve the efficiency and robustness of every aspect of data processing will play a critical role in transforming cryoET from a niche technique into a routine method for in situ structural biology.

## Methods

### Sample preparation

For the minicell samples, JCVI-syn3A cells (J. Craig Venter Institute) were grown at 37 °C without aeration or agitation in SP4-KO medium until reaching an A600 of 0.11. In total, 4 μl of cell supernatant was then applied directly to Quantifoil copper 200-mesh R2/1 grids. For the purified synaptosome samples, hippocampus tissue from 10-week-old rats (Transnetyx) was homogenized by mortar and pestle, followed by 10 min of centrifugation at 800*g* at 4 °C. Samples were diluted in Hibernate A media (Transnetyx) at a ratio of 1:10 or 1:50 before being applied to copper 200-mesh R1.2/1.3 or R2/2 Quantifoil grids. For the affinity-captured lysosomes, a HEK293T cell line with a knock-in mEGFP tag on the C terminus of TMEM192 was used^28^. Cells were lysed in hypotonic homogenization buffer (25 mM Tris-HCL pH 7.5, 50 mM sucrose, 0.2 mM EGTA, 0.5 mM MgCl_2_) and sheared with a 23-gauge syringe. The lysate was equilibrated in sucrose buffer (2.5 M sucrose, 0.2 mM EGTA, 0.5 mM MgCl_2_), and the nuclear fraction was removed by centrifugation at 1,000*g* for 10 min. For the gold-labeled samples, cell lysate was incubated with 0.5–2 μM SidK–HaloTag or SpyCatcher–HaloTag fusion protein conjugated to 2.2-nm or 3-nm maleimide-functionalized gold particles (Nanopartz), respectively. Incubation was performed in a ThermoMixer (Eppendorf) at 4 °C for 1 h with shaking at 500 rpm. Around 6 μl of lysate was then applied to grids functionalized with anti-GFP nanobodies^29^, incubated for 10 s, and washed with 6 μl of phosphate buffered saline (PBS). This sequence was repeated three times, with excess liquid removed by filter paper after each addition and incubation. The grid was then washed with 6 μl of PBS, and the addition of lysate and PBS wash steps were repeated once more. A final 6 μl of PBS was added to the grid. All samples were blotted using a Whitman no. 1 blotting paper for 4–6 min with the chamber conditions set to 4 °C and 95-100% humidity and plunge-frozen in liquid ethane at −180 °C using a Leica GP2.

For the cilia and centrosome lamellae, hTERT RPE-1 cells (American Type Culture Collection, CRL-4000, female) stably expressing HTR6–HaloTag3 (ref. ^30^) were plated at ~20,000 cells per cm^2^ on glow-discharged R2/2 silicone dioxide grids with gold bars (Quantifoil) in a 35-mm glass-bottom dish (20 mm no. 1.5 coverglass, Cellvis) in 10% serum-containing media (DMEM:F12, American Type Culture Collection 30-2006; day 0) at 37 °C in 5% CO_2_. The next day, cells were serum-deprived with 0% FBS media and 100 ng ml^−1^ doxycycline to induce HTR6–HaloTag expression. After 48 h of serum deprivation, HTR6–HaloTag cells were labeled with 250 nM Janelia Fluor 552 dye for 1 h. Cells were rinsed in PBS, incubated for 5 s in HEPES-buffered imaging media (140 mM NaCl, 20 mM HEPES, 2.5 mM KCl, 1.8 mM CaCl_2_, 1.0 mM MgCl_2_, pH 7.4; Live Cell Imaging Solution, Thermo Fisher Scientific or TFS) with 10% glycerol, and plunge-frozen on a Leica EM GP2 plunge freezer with the chamber temperature set to 35 °C and humidity at 95%. Grids were blotted for 3 s. Lamella were prepared using fluorescence-guided milling on an Aquilos (TFS) with an integrated fluorescence microscope.

For the yeast lamellae, a *S. cerevisiae* strain expressing Myo5–GFP, Myo3–GFP and Ab1–RFP fusion proteins were grown to log phase, applied to 200-mesh copper R3.5/1 grids, and plunge-frozen on a Leica GP24. Lamella milling was performed in an Arctic FIB-SEM (TFS) under cryogenic conditions. The TFS WebUI software was used for the entire workflow. Grids were coated with three platinum layers: a sputtering layer (12 kV, 120 nA, 120 s) of metallic platinum, a gas injection system layer (120 s) of organo-platinum and a second sputtering layer (12 kV, 120 nA, 120 s) of metallic platinum. Lamellae were milled in four sequential steps using a 30-kV Xenon ion beam at 1,000 pA, 300 pA, 100 pA and 30 pA for the rough, medium, fine and polishing steps, respectively.

### Data collection

All data were collected on a Krios G4 (TFS) equipped with an X-FEG electron gun, a Falcon 4i detector and the SelectrisX energy filter. The pixel size was set to 1.54 Å (subsequently calibrated to 1.51 Å) for the minicell, synaptosome and lysosome samples; 2.37 Å for the cilia and centrosome lamellae; and 1.94 Å for the yeast lamellae. A total dose of 120 e^−^/Å^2^ was applied for the lysosome, synaptosome and lamella data, and 150 e^−^/Å^2^ was applied for the minicell data. The target defocus was 2 μm for the synaptosome, lysosome, minicell, cilia and centrosome lamellae; and 2.5–4 μm for the yeast lamellae. Data were acquired with Tomography 5 Software (TFS), with beam-image shifts used to collect multiple targets at each stage position. Movies were saved in EER format.

### Self-adaptive defocus search range and step size

AreTomo3 automatically selects the optimal defocus search range and step size based on the magnification change given by equation (1):

1 $$\frac{f}{{f}_{r}}={\left(\frac{p}{{p}_{r}}\right)}^{2}$$

where *f*, *p* and the subscript *r* denote the defocus, pixel size and reference, respectively. Based on a default search range and step size at a reference magnification that yields a pixel size of 1 Å, AreTomo3 rescales these parameters by the square of the corresponding pixel size to accommodate different magnifications.

Equation 1 can be derived as follows. The CTF phase shift, φ, is given by equation (2):

2 $${\rm{\phi }}=0.5{\rm{\pi }}{c}_{s}{{\rm{\gamma }}}^{3}{s}^{4}-{\rm{\pi }}f{\rm{\gamma }}{s}^{2}$$

where *c*_*s*_, γ, *s* and *f* are the spherical aberration coefficient, electron wavelength, spatial frequency and defocus, respectively. Ignoring the contribution of the spherical aberration, which contributes less to the phase shift than the defocus, two CTFs have approximately the same phase shift if equation (3) is satisfied:

3 $${f}_{1}{s}_{1}^{2}={f}_{2}{s}_{2}^{2}$$

The discrete spatial frequency *s*^*(i)*^ can be expressed as follows in equation (4), where *p* denotes the pixel size, and *n* and *N* refer to the *n*th Fourier component and the size of the Fourier transform, respectively:

4 $${s}^{\left(n\right)}=n/\left({pN}\right).$$

Plugging equation (4) into equation (3) gives equation (5):

5 $${f}_{1}{\left(\frac{n}{{p}_{1}N}\right)}^{2}={f}_{2}{\left(\frac{n}{{p}_{2}N}\right)}^{2}$$

which can be simplified to equation (1).

### CTF estimation

AreTomo3 estimates the CTF parameters by jointly optimizing the tilt offsets, tilt axis ambiguity factor (described below) and per-tilt CTF parameters. This is performed by dividing each tilt image into overlapping tiles and computing the power spectrum of each tile. The power spectra are background-subtracted using an approach similar to CTFFIND’s^31^. For the *i*th tilt image, the defocus at the *j*th tile center, *f*_*ij*_, experiences a shift relative to the defocus at the image center, *f*_*i*_, according to equation (6):

6 $$\begin{array}{l}{f}_{i,j}={f}_{i}+{t}_{a}\left[-\left({x}_{{ij}}^{\prime}\cos {\theta}+{y}_{ij}^{\prime}\sin {\theta}\right)\tan \left({\alpha}_{i}+{\alpha}_{0}\right)\right. \\ \left. \qquad\quad+\left({x}_{{ij}}^{{\prime} }\sin {{\theta }}-{y}_{{ij}}^{{\prime} }\cos {{\theta }}\right)\tan {{\upbeta }}_{0}\cos \left({{{\alpha }}}_{i}+{{{\alpha }}}_{0}\right)\right]\end{array}$$

where α_i_ denotes the nominal tilt angle, θ is the tilt axis angle, α_0_ and β_0_ are the sample’s tilt offsets at nominal 0°, *x*′_*ij*_ and *y*′_*ij*_ are the tile’s center coordinates with respect to the raw image coordinate system, and *t*_*a*_ is the tilt axis ambiguity factor. Following the approach in BSoft^18^, the power spectrum of each tile is rescaled based on its local defocus as given by equation (7):

7 $${s}^{{\prime} }=s{\left(\frac{{f}_{{ij}}}{{f}_{i}}\right)}^{1/2}$$

where *s* and *s*′ are the spatial frequencies before and after rescaling, respectively. Spectral rescaling increases the spatial coherence of the tilt image’s average power spectrum by compensating for the defocus gradient. AreTomo3 then jointly optimizes the CTF parameters similarly to the approach taken in Zhang et al.^32^ with the defocus constrained by α_0_, β_0_ and *t*_*a*_ to maximize the objective function as given by equation (8):

8 $$\mathop{\arg \,{\mathrm{max}}}\limits_{{f}_{i},{g}_{i},{\eta }_{i},{\alpha }_{0},{\upbeta }_{0},{t}_{a}}\mathop{\Sigma }\limits_{i}\mathrm{CC}(\overline{{p}_{i}},{\mathrm{CT}{\rm{F}}}_{i}^{2})$$

where $\overline{{p}_{i}}$ is the average of the rescaled power spectra of tiles extracted from tilt image *i*, CTF_*i*_ is the predicted CTF for the tilt image, CC is the cross-correlation coefficient, and *g*_*i*_ and *η*_*i*_ are the magnitude and azimuth angle of astigmatism, respectively. Because the rescaling factor in equation (7) depends on *f*_*i*_, optimization initially estimates this parameter before accounting for the tilting-induced defocus gradient and performing spectral rescaling. The optimal parameter set is found by a multiscale grid search followed by coordinate ascent.

AreTomo3 adopts the coordinate system in which the *z* axis points toward the electron source. The tilt axis ambiguity factor, *t*_*a*_, is determined with respect to this reference frame. Specifically, optimization of the CTF parameters compares *t*_*a*_ = ±1 given the initial estimate of the tilt axis angle (equation (6)). The correct choice enhances the tiles’ spatial coherence during spectral rescaling^18^ and in turn yields a higher cross-correlation between the average power spectrum and the modeled CTF. This effect is most pronounced for tilt images with a large defocus gradient. If *t*_*a*_ is determined to be negative, the tilt axis angle estimated by the common line approach^8^ is corrected by subtracting 180°.

### Sample thickness estimation

Sample thickness is estimated per tilt series by first reconstructing 10×-binned tomograms by the simultaneous algebraic reconstruction technique (SART)^33^ and then computing the cross-correlation between adjacent slices along the tomogram’s depth. These correlation profiles tend to have characteristic shapes, with higher values in the central region due to the continuity of structural information between adjacent *xy* slices within the sample. The correlation decays to minima beyond the sample boundaries before increasing again near the tomogram edges due to backprojection artifacts. AreTomo3 determines the sample boundaries to be positioned where the correlation falls to the value halfway between the local maximum nearest the center of the profile and the minima on either side.

### Local CTF correction

CTF corrections can be broadly categorized into two approaches: phase flipping and Wiener filtering^21^. The former flips the sign of the Fourier components at the frequencies where the CTF is negative, consequently restoring the correct phases while preserving the original noise statistics. However, phase flipping does not compensate for CTF-induced amplitude oscillations, which are most pronounced in the low-to-medium frequency range. By contrast, Wiener filtering aims to correct both the amplitude and phase terms but requires estimating the spectral signal-to-noise ratio (SSNR), which is difficult to do accurately in the low SNR regime. This approach also distorts noise statistics, especially near the zero-crossings where a poorly estimated SSNR dominates the filter. To balance these concerns, AreTomo3 implements a hybrid correction scheme that transitions from a modified Wiener filter at low spatial frequencies to phase flipping at high frequencies, where the filter that adjusts the amplitude oscillations is attenuated. The correction takes the form as given by equation (9):

9 $$O(u,v)=\frac{F(u,v)(1+\gamma )}{|{\rm{CTF}}|+\gamma }{\rm{sign}}({\rm{CTF}}){e}^{-b{s}^{2}}$$

where *O(u,v)* and *F(u,v)* are the Fourier transforms of the image and its underlying object, respectively; *s* is the magnitude of the coordinates *u* and *v* in frequency space; and *γ* is a positive regularization term that increases with spatial frequency so that phase flipping dominates at high resolution. Similar to Warp^19^, a low-pass filter is applied as part of the CTF correction. AreTomo3 applies a default but adjustable B-factor, *b*, of 15 Å^−2^.

To apply this correction locally, each raw tilt image is divided into a series of overlapping and apodized tiles to avoid checkerboard artifacts. The deconvolution is performed on each tile, with all tiles from each tilt image sharing the same per-tilt CTF parameters except for the defocus, which is calculated based on equation (6) to account for its local variation across the tilt image. The CTF-corrected image is then reconstructed by stitching the central squares of adjacent tiles. Analogous to the approach taken in TomoAlign^34^, this strategy of deconvolving the CTF from a region larger than the one used during image reconstruction reduces signal delocalization and CTF aliasing in the high-frequency domain^35^.

### STA

The benchmark dataset^17^ used for STA is available at the CryoET Data Portal^36^ under deposition ID CZCDP-10310; this includes the particle coordinates, tilt series and relevant metadata. The py2rely package (https://github.com/chanzuckerberg/py2rely/) was used to convert the coordinates from copick’s^37^ to RELION-5’s^16^ format and export the global shifts and per-tilt defocus estimates from AreTomo3. STA was then performed as described in Peck et al.^38^. Bayesian polishing and CTF refinement were not performed so that reconstruction quality was based on AreTomo3’s alignments and CTF estimates, unaffected by further refinement from RELION-5. To assess reconstruction quality, the global resolution was estimated based on the Fourier shell correlation between half maps with the mask used during refinement applied to each map. Local resolution was estimated in RELION-5 on the unbinned maps with the sampling rate tuned based on the particle’s diameter.

### Automation of contrast enhancement

When run in parallel with AreTomo3 during live data collection, DenoisET continually monitors the quality metrics file generated by AreTomo3 to track tomograms that meet all user-specified thresholds. The metrics currently considered are sample thickness, deviation of the tilt axis angle from its median value, the maximum global shift, the fraction of bad local patches and the CTF resolution and score at 0°. For processing concurrent with data collection, training begins as soon as sufficient high-quality tomograms are detected. Around 10–40 training tomograms were used for the datasets described in this work. Paired subvolumes of 96 pixels cubed are extracted from the corresponding even and odd tomograms, with data augmentation (consisting of random 90° rotations, mirror operations across the center plane and swapping which subvolume is considered even or odd) applied.

DenoisET implements the Noise2Noise algorithm^23^ for cryoET data, with a similar model architecture as implemented in Topaz-Denoise^26^ but a kernel size of 3 pixels for all layers. In addition to the training and validation loss scores, DenoisET tracks the standard deviation of denoised subvolumes and a metric that quantifies the emergence of checkerboard artifacts during each training epoch. Due to GPU memory constraints, the denoising model is applied to overlapping subvolumes with the padded regions excised during stitching to avoid boundary artifacts. However, empirically we have observed that checkerboard features tend to emerge concurrently with other over-denoising artifacts, such as halos around the perimeter of high-contrast regions and over-suppression of low-contrast features. To quantify this checkerboard artifact, the intensity difference between adjacent pixels in the *xy* planes that form the border between neighboring subvolumes is compared to this value from nearby adjacent planes that lie entirely within each subvolume under consideration. The relative change is computed for each pair of subvolumes with neighboring *xy* planes within the full denoised volume. Border regions for the other planes are not considered to avoid missing wedge artifacts. When the mean of this statistic exceeds a threshold value, DenoisET automatically reloads the model from the prior training epoch and transitions to inference. If the checkerboard metric does not exceed this threshold value during training, DenoisET selects the model from the training epoch associated with the highest standard deviation for the denoised subvolumes and applies that during inference. For real-time processing, DenoisET continually checks for new tomograms to denoise.

### Evaluation of contrast enhancement

The impact of AreTomo3’s CTF correction on denoising was assessed by comparison with the deconvolution implemented in IsoNet^22^, which is based on the approach taken in Warp^19^. The IsoNet CTF deconvolution was applied to weighted backprojection tomograms reconstructed by AreTomo3 using the defocus value estimated at 0°. A grid search was performed to determine optimal values for the fall-off and strength parameters in the SSNR term of the Wiener-like filter. A denoising model was trained separately on each set of tomograms, and the combination of values that led to the most effective denoising based on visual inspection was chosen. Empirically we found that denoising was more sensitive to the fall-off than the strength parameter. No tuning of the B-factor that adjusts the strength of AreTomo3’s CTF deconvolution was performed for any of the datasets presented in this work. A separate denoising model was trained on the AreTomo3-deconvolved tomograms.

Contrast enhancement was quantified by computing the ratio of the power spectra of regions of interest to background regions for different tomogram types. The regions of interest consisted of 224 virus-like-particles identified across 45 tomograms, while 135 background regions were selected from the same set of tomograms based on local intensity statistics. Pixel subvolumes of 80^3^ units centered on each region of interest or background region were extracted from tomograms reconstructed with a pixel size of 5 Å. A cosine mask was applied to each subvolume in real space before computing the radially averaged power spectrum. The ratio of the mean power spectra of the regions of interest and background regions was then calculated. Comparing regions of interest and background from the same tomogram type provides an estimate of the inherent contrast of localized regions that would be targeted by downstream analysis such as particle picking. Importantly, this ratio only increases if contrast enhancement is more pronounced in the regions of interest; strategies that uniformly increase contrast throughout the tomogram will not affect this ratio. Supplementary Note 4 provides a qualitative comparison of DenoisET to other denoising schemes.

### Reporting summary

Further information on research design is available in the Nature Portfolio Reporting Summary linked to this article.

## Online content

Any methods, additional references, Nature Portfolio reporting summaries, source data, extended data, supplementary information, acknowledgements, peer review information; details of author contributions and competing interests; and statements of data and code availability are available at 10.1038/s41592-026-03093-y.

## Supplementary information

Supplementary Information Supplementary Notes 1–4.

Reporting Summary

## Figures and Tables

**Extended Data Table 1:**
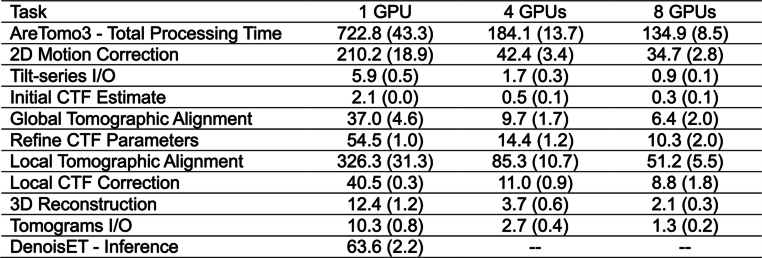
AreTomoLive’s performance

Performance was measured on the indicated number of NVIDIA RTX A6000 GPUs. The time required to complete each processing task per tilt-series is normalized by the number of GPUs and listed in seconds, with the standard deviation from processing more than 100 tilt-series noted in parenthesis. The raw data were collected at a pixel size of 1.51 Å, image dimensions of 4096 ***×*** 4096 pixels, and 31 tilt images per tilt-series. 2D motion correction with 4*×*4 local patches was performed on super-resolution EER movies, each containing 170 or 225 frames, followed by downsampling to the original pixel size before performing tomographic alignment with 4*×*4 patches. CTF-corrected even, odd, and full tomograms were reconstructed at a pixel size of 5 Å and a volume depth of 250 nm. Nearly linear scaling was observed on 4 GPUs, with a slight reduction on 8 GPUs due to increased overhead. Currently, DenoisET does not support parallelization. Runtime comparisons to other software packages are provided in Supplemental Note 2.

## Data Availability

The tomograms from the minicell, synaptosome, lysosome and annotation benchmark datasets are available on the CryoET Data Portal under deposition IDs CZCDP-10312, CZCDP-10313, CZCDP-10318 and CZCDP-10310, respectively.

## Code availability

AreTomo3 is available at https://github.com/czimaginginstitute/AreTomo3/. DenoisET is available at https://github.com/apeck12/denoiset/. The versions of the code described herein and instructions that demonstrate using this software on a dataset from the CryoET Data Portal are available on Zenodo via 10.5281/zenodo.19352241 (ref. ^39^).
