# Supplementary material for: AreTomoLive: automated reconstruction of comprehensively corrected and denoised cryo-electron tomograms in real time and at high throughput
Source: Nat Methods. 2026 May 25;23(6):1121–5. doi: 10.1038/s41592-026-03093-y (PMC13259924; doi:10.1038/s41592-026-03093-y)
Supplement: Supplementary file 1 — Supplementary Notes 1–4. [file 41592_2026_3093_MOESM1_ESM.pdf]

nature methods

Supplementary information https://doi.org/10.1038/s41592-026-03093-y

AreTomoLive: automated reconstruction of comprehensively corrected and denoised cryo-electron tomograms in real time and at high throughput

In the format provided by the authors and unedited

Contents

Supplemental Note 1: Tomogram curation based on AreTomo3's quality metrics **2**

Supplemental Note 2: Runtime comparisons between AreTomo3 and other preprocessing pipelines **6**

Supplemental Note 3: Validation of AreTomo3's CTF measurement **8**

Supplemental Note 4: Comparison of DenoisET to established denoising methods **10**

References **13**

Supplemental Note 1: Tomogram curation based on AreTomo3's quality metrics

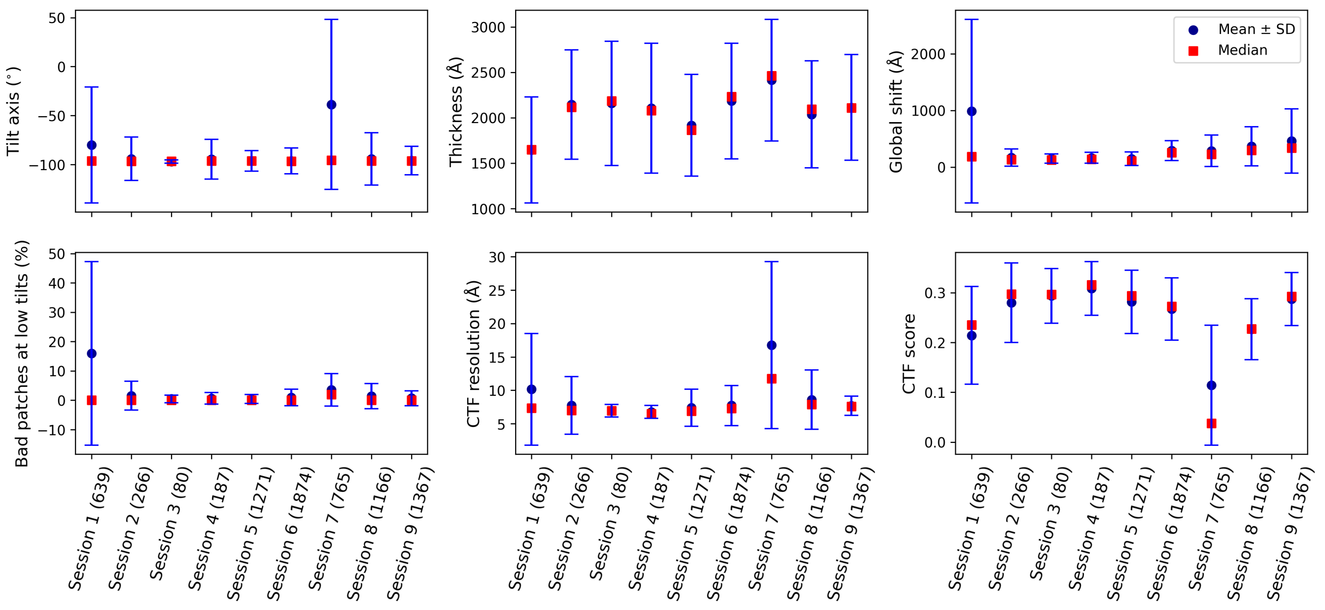

Supplemental Figure 1: Comparison of AreTomo3's metrics across data collection sessions from similar samples and with the same acquisition parameters for quality monitoring. AreTomo3's metrics are compared across nine datasets acquired from affinity-captured lysosome samples using identical optical settings and acquisition schemes. The number of tilt-series in each dataset is indicated in parenthesis. The error bars indicate the standard deviation. Outliers, particularly in the estimated tilt axis angle and CTF scores, often indicate low-quality data.

Every step of the cryoET workflow is susceptible to errors, which can propagate to and impair downstream steps. AreTomo3 provides a screening mechanism to exclude some low-quality data from subsequent processing. For each reconstruction, AreTomo3 records six metrics, spanning tomographic alignment, information transfer, and sample geometry. For alignment, it reports the tilt axis angle, maximum global shift, and the fraction of patches with failed local alignment. For information transfer, it provides the Thon ring resolution and CTF estimation score for the 0° image. Sample thickness is reported for geometry, though the reliability of this measurement depends on a successful initial alignment. Anomalous thickness estimates can thus be a useful diagnostic that indicate failures during upstream alignment. For datasets collected from similar samples and with the same acquisition parameters, comparing these metrics offers insights into dataset quality (Supplemental Fig. 1). In addition to quality monitoring, these metrics can be leveraged for tomogram curation.

The effectiveness of applying k-means clustering to these metrics as a screening mechanism was evaluated by comparing its classification to visual inspection by expert annotators. A dataset of 789 tomograms of *Mycoplasma mycoides* JCVI-Syn3A "near-minimal" cells[1] (minicells) was reviewed, with 245 high-quality tomograms retained by users for STA (Supplemental Fig. 2a-b). K-means was then applied to independently classify the tomograms into two clusters. The cluster (Supplemental Fig. 2c-h, cluster 0) with a higher standard deviation across all metrics was considered the rejected class. Compared to visual inspection, k-means clustering correctly classified 61% of user-rejected tomograms. Only 8% of user-selected tomograms was misclassified, even though a criterion during manual screening was ribosome abundance, which AreTomo3's quality metrics do not directly report on.

By comparison, a constant thresholding approach that classified tomograms based on specific cut-off values for all six of the reported metrics correctly classified 77% of the user-rejected tomograms but incorrectly rejected 26% of the user-selected tomograms, more than three times the rate of incorrect rejections of the k-means approach (Supplemental Fig. 3). The thresholds used for these metrics were largely chosen based on the dataset's statistics in an effort to remove outliers. Though tuning the cut-off values might improve consistency with the results of visual inspection, such per-dataset adjustments are subjective and reduce the generalizability of this approach. To evaluate the generalizability of k-means clustering, this analysis was repeated for a benchmark dataset for annotation algorithms[2] and a dataset of affinity-captured lysosomes (Supplemental Fig. 4). In these cases, k-means clustering

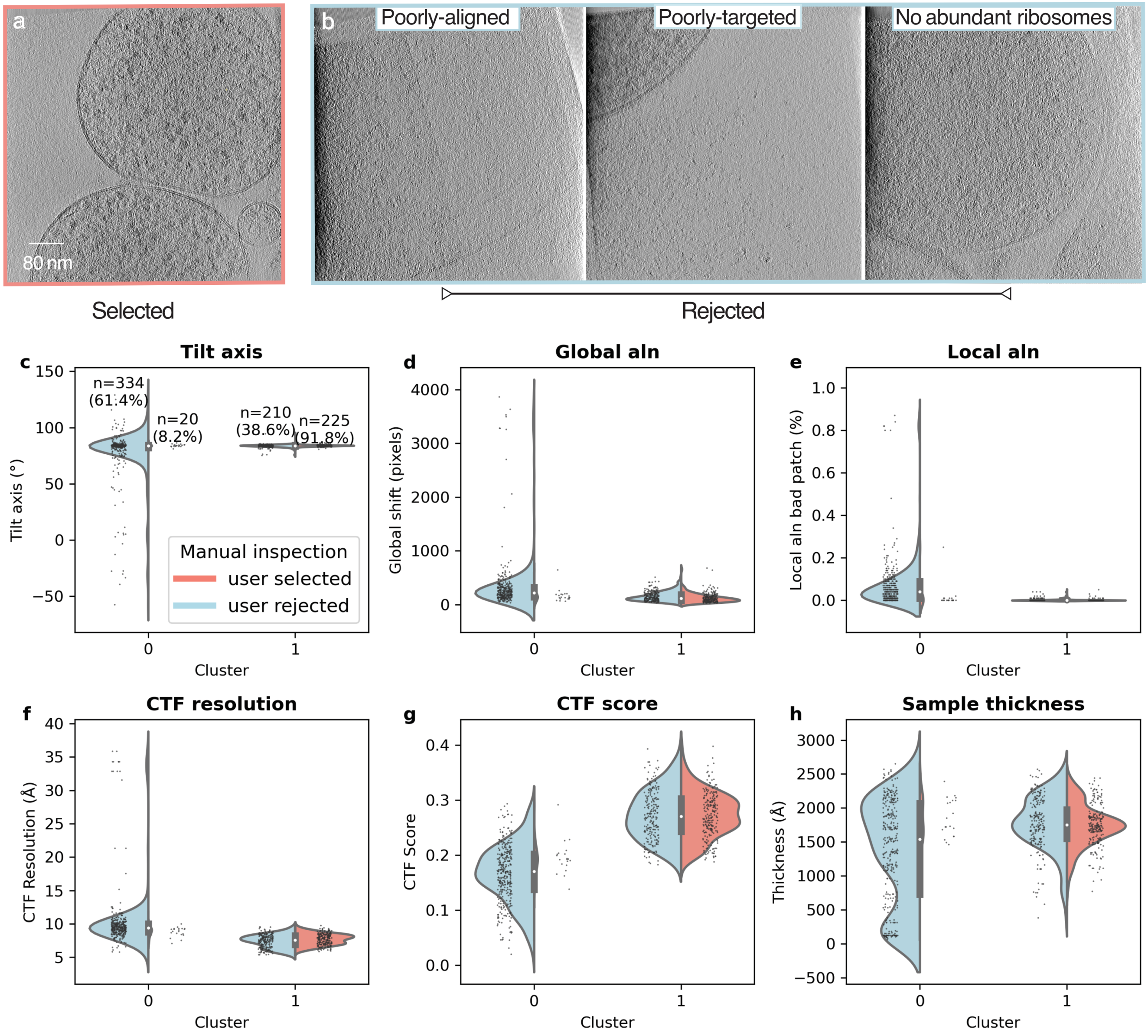

Supplemental Figure 2: Visual inspection versus k-means clustering of AreTomo3's metrics applied to a dataset of minicell tomograms. During visual inspection, **a.** well-aligned tomograms with clearly discernible ribosomes were selected while **b.** tomograms that were poorly-aligned, a result of mistargeting, and/or lacking abundant ribosomes were rejected. 245 of 789 tomograms were selected for downstream analysis based on these criteria. K-means clustering with 2 clusters was performed on AreTomo3's metrics. The resulting clusters are color-coded by user annotation and plotted as violin plots with scattered data points overlaid for the following metrics: **c.** tilt axis angle, **d.** maximum shift during global alignment (aln), **e.** percentage of patches with failed local alignment, **f.** Thon ring resolution, **g.** CTF estimation score for the zero-tilt image, and **h.** sample thickness. The number and percentage of user-rejected (blue) and user-selected (red) tomograms in each cluster are reported in panel **c.**, with percentages calculated relative to the total user-rejected (544) and user-selected (245) tomograms. Compared to visual inspection, k-means clustering as a screening mechanism successfully classified over 61% of the user-rejected tomograms (334 out of 544) while misclassifying ∼8% of the user-selected tomograms (20 out of 245).

correctly classified 45–53% of the user-rejected cases into the rejected cluster for these datasets, with 13-25% of the user-selected tomograms misclassified. The reduced consistency between k-means clustering and user annotations for these datasets compared to the minicell dataset may reflect the more biologically-driven selection criteria that

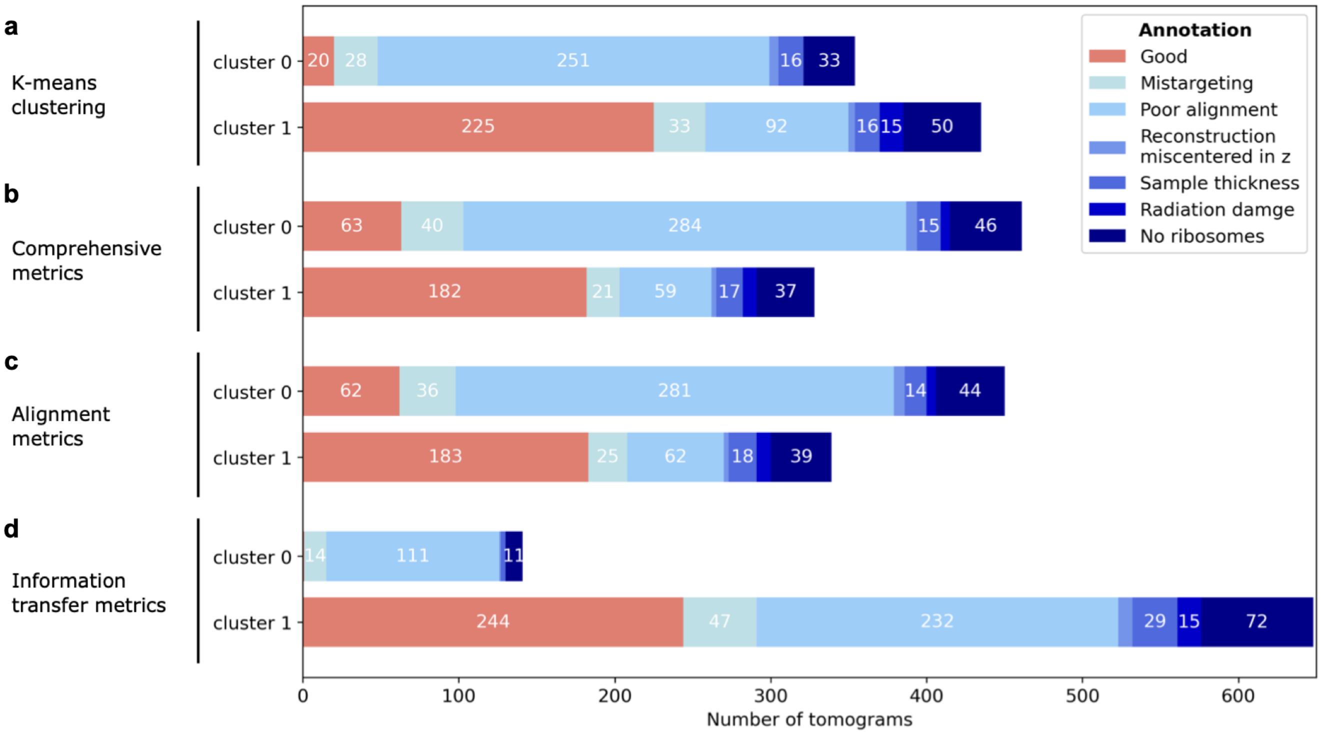

Supplemental Figure 3: Comparative analysis of k-means clustering and a constant thresholding approach of AreTomo3's metrics for classifying tomograms. Clustering results are compared for different classification strategies applied to a large dataset of minicells. **a.** K-means clustering with 2 clusters was applied, as shown in Fig. 2. **b-d.** Classification was performed based on constant thresholds. The following thresholds were applied as criteria for rejecting tomograms: (i) the estimated tilt axis orientation deviating by more than 0.75° from the dataset's median; (ii) sample thickness exceeding one standard deviation above the median (∼360 nm); (iii) the maximum global shift exceeding 10% of the tilt series x/y dimensions (∼630 Å); (iv) patches classified as failures during local alignment exceeding 5% for tilts within ±30° and more than 10% across the full tilt range; and (v) CTF correlation score or (vi) CTF resolution worse than one standard deviation from the median. Classification results are shown based on **b.** all six criteria, **c.** only alignment metrics (i, iii, iv), and **d.** only the information transfer metrics (v, vi).

guided visual inspection, or the fact that the tomograms used for k-means clustering and visual inspection were reconstructed by different software packages. Regardless, the ability to automatically discard half of low-quality tomograms would significantly reduce the time spent on manual curation for large datasets.

Compared to applying constant thresholds, this k-means clustering approach partitions tomograms more similarly to expert annotations and requires minimal parameter tuning from one dataset to the next. In contrast to supervised deep learning methods, this unsupervised approach avoids the need for large manually-annotated datasets[3], though in the future it will be useful to see how this approach compares to recent neural network-based approaches to score tomogram quality[4]. Including additional metrics — in particular, the α_0 and β_0 offsets for lamella samples — and applying different clustering methods may further improve the effectiveness of this screening strategy.

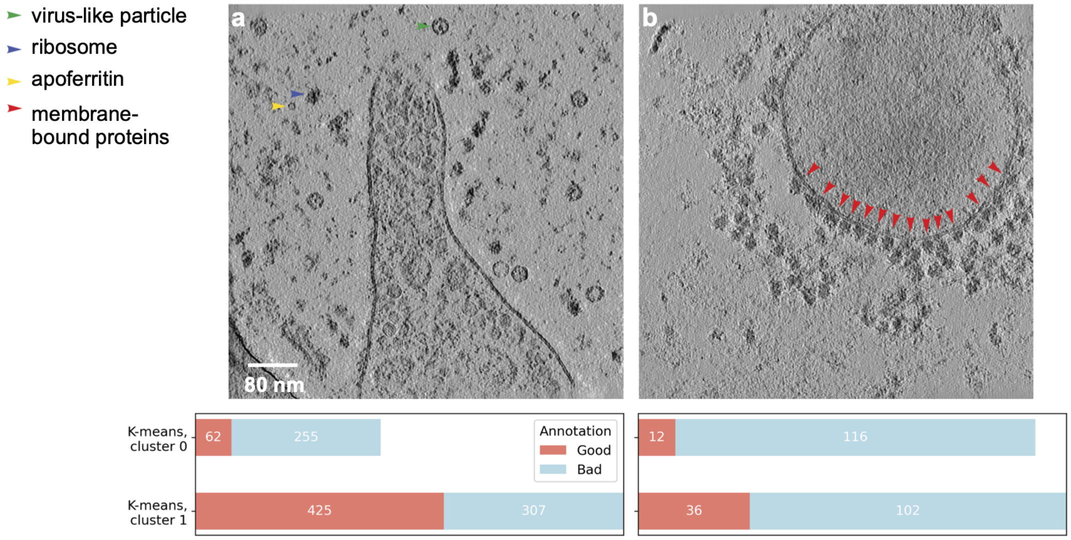

Supplemental Figure 4: **Comparison of k-means clustering on AreTomo3's metrics and user classification of a benchmark dataset for annotation algorithms[2] and a dataset of affinity-purified lysosome.** **a.** For the benchmark dataset, users selected tomograms that contained sufficient copies of the proteins of interest (three examples annotated) and appeared well-aligned. 45% (255 of 562) of user-rejected tomograms was captured by the rejected cluster in k-means clustering (cluster 0), while 13% (62 of 487) of user-selected tomograms was misclassified. As quality metrics were introduced as a feature after visual inspection was performed, the tomograms used for visual inspection and clustering were reconstructed by different version of AreTomo3. **b.** In the affinity-captured lysosome dataset (266 tomograms), users selected tomograms based on alignment quality and the presence of membrane-bound proteins (red arrows). Visual inspection was performed on tomograms reconstructed using Athena (ThermoFisher Scientific). Among the user-rejected tomograms, 53% (116 of 218) were correctly identified by clustering, while 25% (12 out of 48) of user-selected tomograms were misclassified. The higher misclassification rate likely reflects that AreTomo3's metrics do not report on the presence of membrane-bound proteins and that user selection was based on tomograms reconstructed by a different software package.

Supplemental Note 2: Runtime comparisons between AreTomo3 and other preprocessing pipelines

<table>
<thead>
<tr><th>Task</th><th>AreTomo3</th><th>nextPYP[1]</th><th>WarpTools[2]</th></tr>
</thead>
<tbody>
<tr><td>Total Time[3]</td><td>691</td><td>1391</td><td>373</td></tr>
<tr><td>2D Motion Correction</td><td>228</td><td>132</td><td rowspan="2">200</td></tr>
<tr><td>Initial CTF Estimation</td><td>2</td><td>1</td></tr>
<tr><td>Tomographic Alignment</td><td>341</td><td rowspan="2">624</td><td>54</td></tr>
<tr><td>CTF Refinement</td><td>35</td><td>18</td></tr>
<tr><td>3D Reconstruction</td><td>23</td><td>37</td><td rowspan="2">98</td></tr>
<tr><td>CTF Correction[4]</td><td>40</td><td>N/A</td></tr>
<tr><td>Gold Detection[5]</td><td>N/A</td><td>477</td><td>N/A</td></tr>
</tbody>
</table>

Supplemental Table 1: **Runtime comparison for tilt-series processing.** Times are reported in seconds and correspond to the processing time averaged across 20 tilt-series by each software package on one NVIDIA RTX A6000 GPU. [1]nextPYP reports the aggregate time required for tomographic alignment and CTF refinement. [2]WarpTools provides a combined function to perform 2D motion correction and initial CTF estimation. [3]The total time includes overhead steps and thus exceeds the sum of the listed tasks. nextPYP also performed a single pre-processing step that took 221 seconds; this is a one-time cost rather than performed per tilt-series and so was not included in the overall time. [4]WarpTools and AreTomo3 optionally perform CTF correction during 3D reconstruction, and AreTomo3 reports these times separately. nextPYP does not have an option to generate CTF-corrected tomograms. [5]Currently gold detection is automatically performed in nextPYP even when a fiducial-less alignment routine is selected.

To benchmark processing speeds, AreTomo3 was compared to nextPYP[5] v0.7.0 and WarpTools[6] v2.0.0. This comparison was performed on one NVIDIA RTX A6000 GPU since all programs scale with the number of allocated resources and using 20 tilt-series from a benchmark dataset for multi-class annotation algorithms[2]. The raw EER movies contained 198 frames, and each tilt-series contained 31 tilt images of dimensions 4096×4096 pixels collected by a dose-symmetric tilt-scheme at a pixel size of 1.51 Å. Reprocessing in AreTomo3 was carried out using the same parameters as previously described[2]. Specifically, motion correction used 4×4 local patches on the super-resolution frames grouped into batches of 10 frames followed by downsampling back to the original pixel size. Tilt-series alignment also used 4×4 local patches. Tomograms were CTF-deconvolved and reconstructed by weighted backprojection with a pixel size of 5 Å and a z-depth of 1200 unbinned pixels. For the purpose of this comparison, odd and even tomograms were not generated.

While both nextPYP and WarpTools provide pipelines that extend to STA, we benchmarked processing times from motion correction of the raw tilt-series to tomogram reconstruction for comparison with AreTomo3. The nextPYP platform stitches together multiple third-party software packages to provide both real-time and offline data processing[5]. For the fairest comparison, we chose GPU-accelerated packages when available, resulting in a processing pipeline that used MotionCor3, CTFFIND4, and AreTomo2 respectively for 2D motion correction, CTF estimation, and tomographic alignment and reconstruction. Tomograms were reconstructed by SART at a pixel size of 4.62 Å (bin 3), as nextPYP does not permit non-integer binning.

WarpTools is the Linux adaptation of Warp/M[6,7] and currently does not provide live processing. For this package, the parameters for 2D motion correction were set to mimic the strategy used in AreTomo3 so that alignment would be performed on regions with equivalent SNRs. Specifically, the EER frames were rendered in groups of 10, and 2D motion correction was performed using a motion model grid with 4×4 local patches in the dimensions of each frame and one grid point per rendered frame. Default parameters were used for the remaining steps, including CTF estimation/refinement and tilt-series alignment using Etomo[8]. CTF-deconvolved tomograms were reconstructed at a pixel size of 5 Å and a z-depth of 1200 unbinned pixels to match the AreTomo3 reconstructions.

Supplemental Table 1 compares the overall runtimes for tilt-series processing and provides a breakdown for the principle component tasks. AreTomo3 provides a 2x overall speed-up compared to nextPYP, which performs an obligatory gold detection step even when fiducial-less alignment is performed. Excluding this task's contribution to the overall time, AreTomo3 is still ∼1.3x faster than nextPYP despite performing a local CTF deconvolution step not available in nextPYP. This difference highlights the advantage of an integrated pipeline that retains data in memory during processing to reduce the overhead of disk I/O. Compared to WarpTools, AreTomo3 was ∼1.9x slower at processing tilt-series, with the time spent on tomographic alignment accounting for most of this difference. Visual comparison of tomograms reconstructed by the three pipelines showed similar alignments but

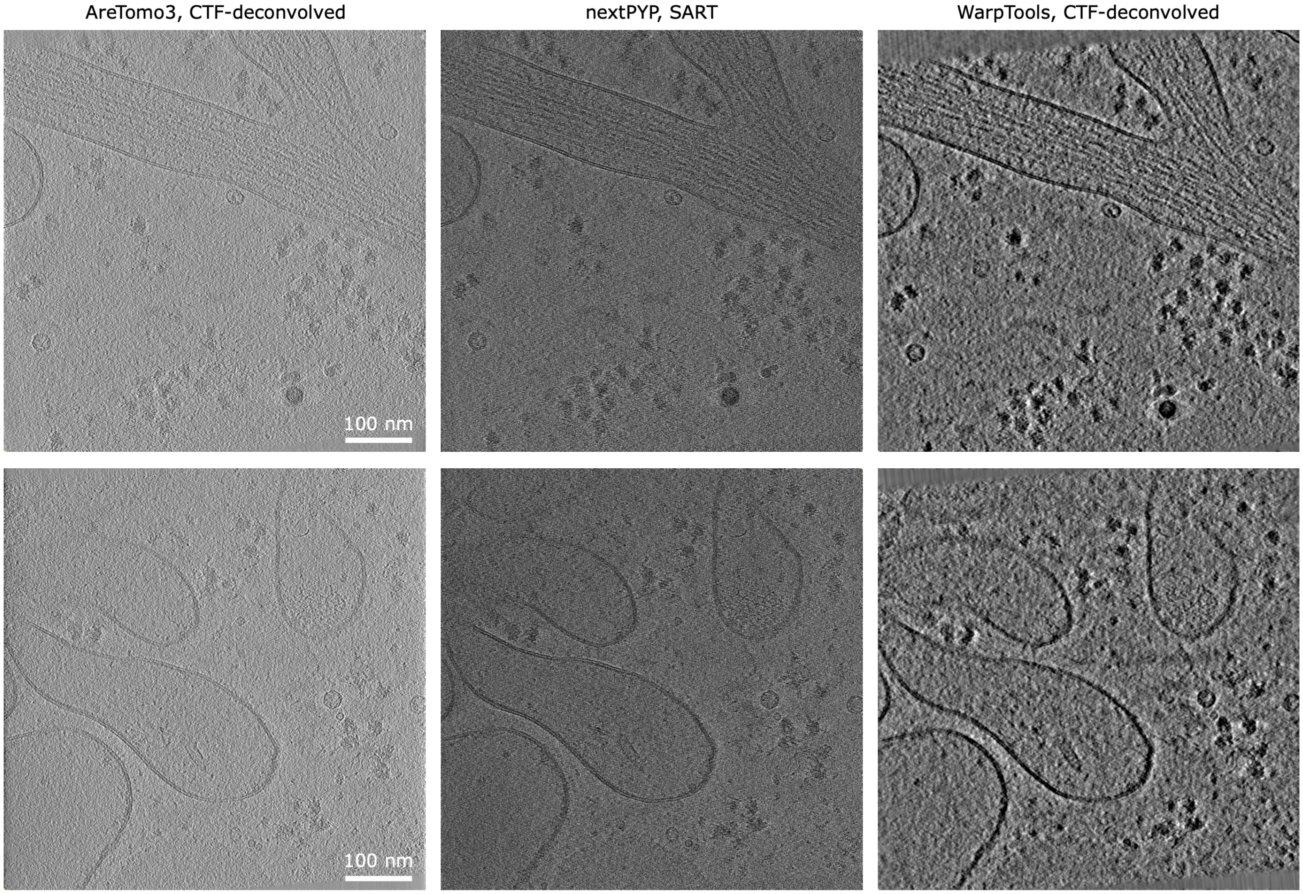

Supplemental Figure 5: **Comparison of tomograms reconstructed by AreTomo3, nextPYP, and WarpTools.** The corresponding slices (5 Å for AreTomo3 and WarpTools and 4.6 Å for nextPYP) for two tomograms are shown.

noticeable differences in contrast (Supplemental Fig. 5). The stronger filter applied by WarpTools compared to AreTomo3's CTF deconvolution yielded halos around high contrast features; the difference in filter strength and its impact are discussed in the main text. The nextPYP-reconstructed tomograms also differed in contrast due to reconstruction by SART rather than weighted backprojection with CTF deconvolution.

Supplemental Note 3: Validation of AreTomo3's CTF measurement

AreTomo3's defocus estimates were compared to those from WarpTools[6] v.2.0.0 and CTFFIND5[9] v.5.0.2. This comparison was performed on a benchmark dataset[2] (CZCDP-10310) of 492 tilt-series collected at a target defocus of 2 μm, a pixel size of 1.51 Å, and a total dose of 62.9 e^-/A^2 linearly spread across the tilt images. Each tilt-series consisted of 31 tilt images spanning ±45° separated by 3° increments and was collected according to a dose-symmetric tilt scheme. WarpTools' CTF estimation was carried out with default parameters, yielding two sets of estimates: the initial defocus measurements after motion correction but prior to tilt-series alignment and final measurements after taking into account the tilt-series geometry. CTFFIND5 was run with the defocus search range set to 1-3.5 μm with a step size of 100 Å; the resolution range used for the parameter search was 5-50 Å. Tilt and thickness estimation were not performed. AreTomo3 automates the defocus search range and step size as described in the Methods. For all three software packages, the input data were the motion-corrected tilt stacks generated by AreTomo3.

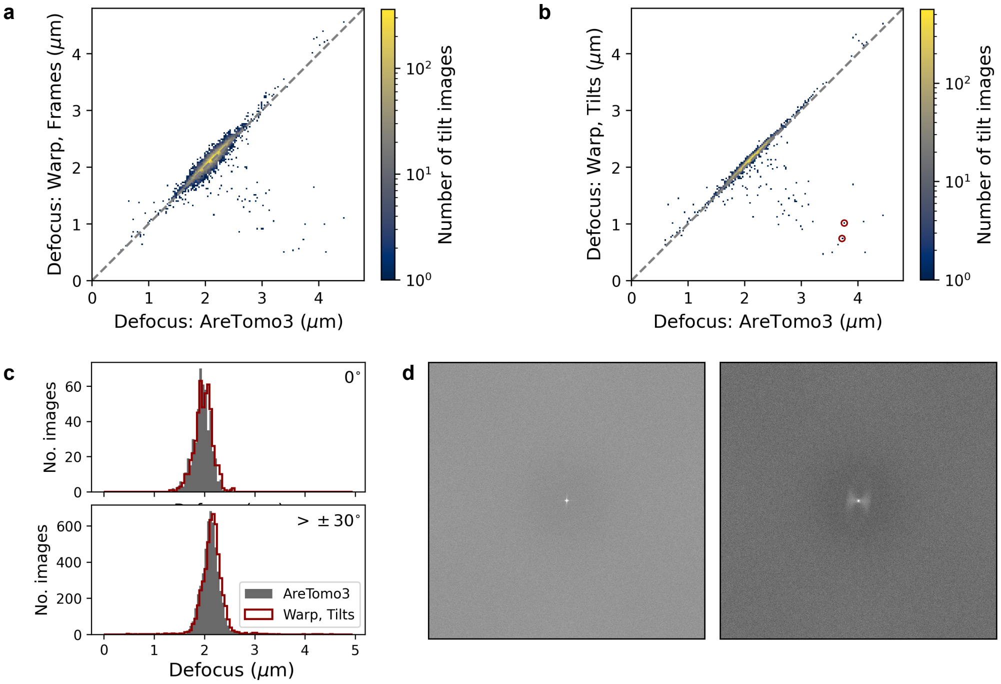

Supplemental Figure 6: Comparison of AreTomo3's and WarpTools' defocus estimates. Defocus estimates from AreTomo3 are compared to the estimates from WarpTools **a** before (Frames) and **b** after (Tilts) taking into account the tilt-series geometry. The tilt images shown in panel d are circled in red in panel b. **c.** The distribution of defocus values for the 0° images (upper) and tilt images collected beyond ±30° (lower) are shown for AreTomo3 (grey) and WarpTools (red). **d.** The Fourier transforms from two tilt images with a large discrepancy in the estimated defocus values between AreTomo3 and WarpTools are visualized.

As shown in Supplemental Fig. 6, the defocus estimates between AreTomo3 and WarpTools showed very good agreement. As expected, this consistency was higher when comparing AreTomo3's refined values and WarpTools' final defocus estimates, which take into account the tilt-series geometry, compared to WarpTools' initial estimates that are based on the motion-corrected frames (Supplemental Fig. 6a-b). While 8.1% of tilt images were characterized by a >0.1 μm difference in defocus between AreTomo3's refined and Warp's initial estimates, this was true for <1% of tilt images when the comparison was performed with Warp's final estimates. The high agreement between AreTomo3's and WarpTools' final defocus estimates was observed even at high tilt angles, with only 1.7% of images collected at high tilt angles (beyond ±30°) characterized by a >0.1 μm defocus difference between AreTomo3's and WarpTools' refined defocus estimates (Supplemental Fig. 6c). Visual inspection of two tilt images with a large difference in the estimated defocus showed no visible Thon rings in the Fourier transform, making CTF fitting unreliable for these images (Supplemental Fig. 6d).

On the other hand, there was poorer agreement between AreTomo3's and CTFFIND5's defocus measurements

(Supplemental Fig. 7a). 30.4% of all tilt images were characterized by a >0.1 μm difference in the estimated defocus; for the tilt images collected at high tilt angles (beyond $\pm 30°$), this increased to 64.1% (Supplemental Fig. 7b). Visual inspection of representative images with a large discrepancy between AreTomo3's and CTFFIND5's defocus estimates suggested that CTFFIND5 was unable to accurately model the CTF in these cases that AreTomo3 and WarpTools successfully measured (Supplemental Fig. 7c).

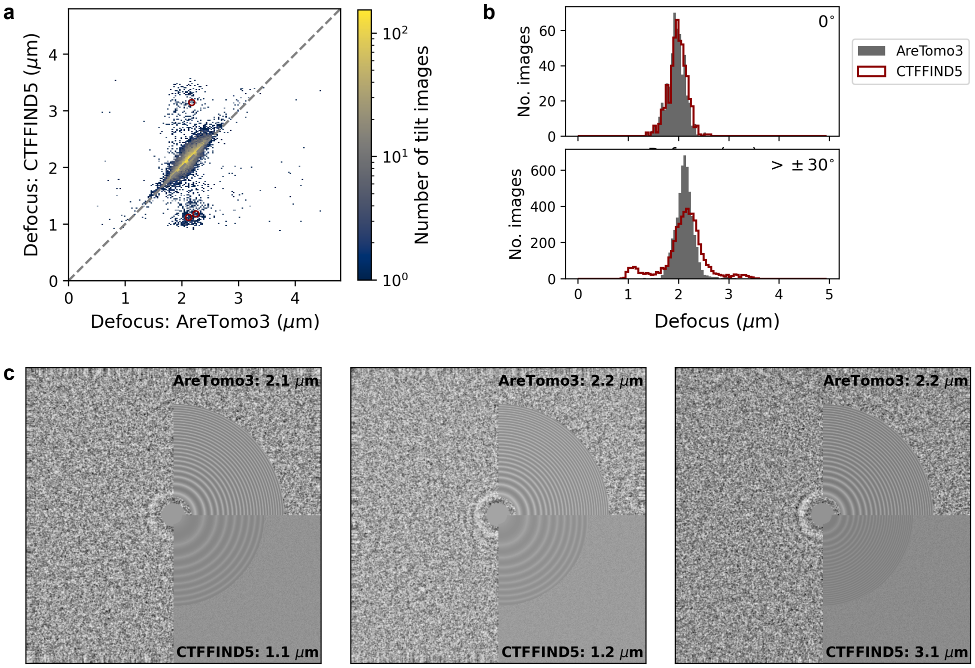

Supplemental Figure 7: **Comparison of AreTomo3's and CTFFIND5's defocus estimates. a.** Defocus estimates from AreTomo3 and CTFFIND5 are compared. The representative tilt images shown in panel c are circled in red. **b.** The distributions of defocus values for the untilted images (upper) and tilt images collected beyond $\pm 30°$ (lower) are shown for AreTomo3 (grey) and CTFFIND5 (red). **c.** Three representative tilt images with discrepancies in the estimated defocus values between AreTomo3 and CTFFIND5 were inspected. The left half of each subpanel shows the radially-averaged power spectrum after background subtraction, as output by AreTomo3 as a diagnostic during CTF measurement. The upper right and lower right quadrants shows AreTomo3's and CTFFIND5's CTF fits, respectively. The left and center images were collected at 45°, while the rightmost image was collected at -42°. The refined defocus estimates from WarpTools for these tilt images from left to right are 2.0, 2.3, and 2.2 μm.

Supplemental Note 4: Comparison of DenoisET to established denoising methods

Contrast enhancement by DenoisET was compared to three established denoising approaches for cryoET data: cryoCARE[10], which was the first implementation of the Noise2Noise algorithm[11] for cryoET data; IsoNet[12], which performs missing wedge restoration and denoising based on the Noisier2Noise algorithm[13]; and DeepDeWedge[14], which carries out simultaneous missing wedge reconstruction and denoising based on the Noise2Noise algorithm. This comparison was made using a dataset of purified synaptosomes that contained biological features across a range of length scales and was both more crowded and characterized by lower SNR than the benchmark dataset[2] used for the comparisons presented in Supplemental Notes 2 and 3. This dataset, which consists of 76 tilt-series, was processed by AreTomo3 to generate paired and full tomograms by weighted backprojection with an optional CTF deconvolution applied at a pixel size of 5 Å.

This dataset was curated to exclude tomograms with a tilt-axis measurement >1° from the dataset's median value, a maximum global shift >400 Å, CTF resolution >10 Å, CTF score <0.2, > 1% failed local patches at low tilt angles, >5% failed local patches across the full tilt-range, and/or a thickness <100 nm. This curation strategy yielded 26 high-quality tomograms for training. The denoising methods were then applied to the reconstructed tomograms as described below:

- DenoisET was run with default parameters. During training, 250 paired subvolumes were extracted from the CTF-deconvolved tomograms for a maximum of 20 epochs. Training was automatically terminated after 13 epochs when the checkerboard metric exceeded the threshold value of 0.034 (see Methods), and the model from epoch 12 was used to perform inference.

- CryoCARE[10] was trained on the same curated set of high-quality CTF-deconvolved tomograms used to train DenoisET. Training and inference were performed using default parameters.

- For IsoNet[12], a subset of five tomograms from the curated set used to train DenoisET and cryoCARE was used since the developers recommend using 1-5 tomograms for training. Training was performed on the weighted backprojection tomograms without CTF correction generated by AreTomo3 since IsoNet applies a distinct CTF deconvolution strategy during preprocessing. AreTomo3's defocus estimates for the 0° image were used for this deconvolution. Pre-processing, training, and inference were performed using default parameters, with the exception of the SNR fall-off parameter that tunes the deconvolution strength. For this, values of both 0.5 and 1.0 (the default value) were tested. The latter yielded superior contrast enhancement, and those results are shown below.

- The performance of DeepDeWedge was demonstrated on datasets ranging from 1-7 tilt-series in size[14], so we trained this model on the same subset of five tomograms used to evaluate IsoNet. DeepDeWedge does not provide its own CTF correction routine, so the CTF-deconvolved tomograms generated by AreTomo3 were used for training. Pre-processing (which includes mask generation in IsoNet and subtomogram extraction), training, and inference were carried out using default parameters with the following exceptions. The missing wedge angle parameter was updated to reflect the ±45° tilt-range of the data, and training was terminated after 50 epochs because the fitting and validation losses had plateaued. The refined model from the final epoch was used for inference.

Task	DenoisET	CryoCARE	IsoNet	DeepDeWedge
Training	350	647	500	1430
Inference (per tomogram)	1	4	10	70

Supplemental Table 2: **Runtime comparison of different denoising approaches.** Times are reported in minutes when the above approaches were run on one NVIDIA A40 GPU. The time required for pre-processing, which includes mask generation for IsoNet and DeepDeWedge and CTF deconvolution for IsoNet, are not included.

Supplemental Table 2 compares the runtimes for each approach. The training times are not directly comparable since DenoisET is the only method that automates the termination of training and transition to inference, while the other methods run for the specified number of epochs. Further, IsoNet and DeepDeWedge were trained on a dataset less than one fifth of the size used to train DenoisET and cryoCARE, following the recommended training procedures for these methods. Even with those caveats, DenoisET provided the fastest training and inference of the four methods, in line with its aim of performing real-time contrast enhancement.

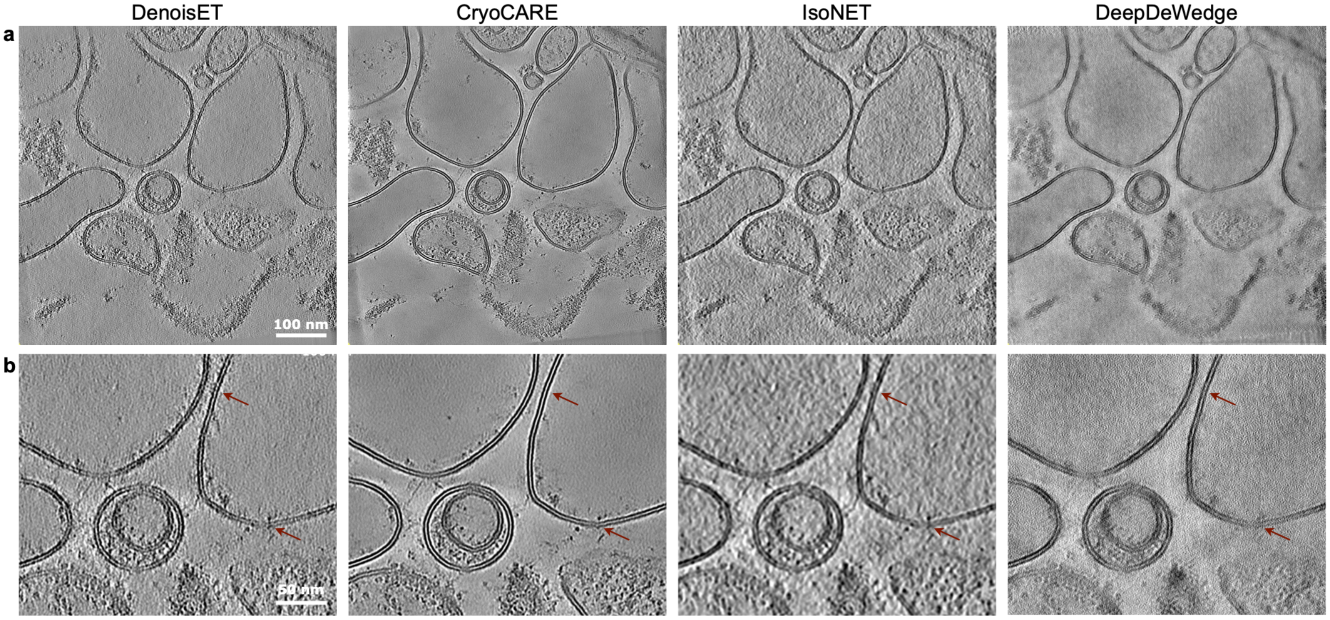

Supplemental Figure 8: **Comparison of denoising approaches on a tomogram from the training set. a.** A slice through the denoised tomogram processed by the indicated software program is visualized. **b.** A central region from the slice shown in panel **a.** is visualized, with arrows highlighting membrane proteins that are differentially retained or suppressed by different denoising schemes.

Supplemental Fig. 8 compares denoising by the four approaches on a volume included in the training set. Visually, DenoisET and cryoCARE provided superior contrast enhancement. Though the solvent regions in the cryoCARE-denoised tomogram appear flatter, this yielded over-suppression of trans- and inter-membrane features compared to the DenoisET-denoised tomogram (Supplemental Fig. 8b). By contrast, the IsoNet- and DeepDeWedge-denoised tomograms appear under- and over-denoised, respectively. It is possible that the poorer performance of algorithms that perform missing wedge restoration is due to the tilt-range ($\pm 45°$) of the synaptosome data, which is narrower by 18-40° than the tilt-range of the datasets those software packages were initially benchmarked against[12,14]. Although this may make missing wedge restoration more difficult, the limited contribution of information at high tilt angles to particle reconstruction[15] has motivated collection schemes that concentrate dose across a narrower range.

Denoising quality was also compared on a tomogram not included in the training data (Supplemental Fig. 9a). Similar trends were observed as for the representative volume from the training set, suggesting that none of the approaches was overfit to the training data. The impact of these denoising approaches on membrane segmentation quality was also assessed based on predictions by the generalizable segmentation model from MemBrain-seg[16]. Overall, the cryoCARE- and IsoNet-denoised tomograms appeared to yield the best membrane segmentation in terms of coverage, with the former resulting in more clearly resolved individual leaflets (Supplemental Fig. 9b, regions 1 and 2). The DenoisET-denoised tomogram yielded better-resolved leaflets than the IsoNet-denoised tomogram but missing predictions for part of the multi-lamellar vesicle and in lower-contrast regions. By contrast, the blurred features in the DeepDeWedge-denoised tomogram resulted in many membranes left undetected (Supplemental Fig. 9b, regions 1 and 3). This comparison is limited to visual inspection due to the lack of ground truth annotations for this dataset. We also note that the pretrained MemBrain-seg model was trained on 10 Å data, in contrast to the 5 Å data used here.

The contrasting results shown in Supplemental Figs. 8-9 underscore the fact that different degrees of contrast enhancement may be desired depending on the downstream task. While the cryoCARE-denoised tomograms yielded the most robust membrane segmentation of the four methods compared here, the DenoisET-denoised tomograms better preserved lower contrast features in the membrane vicinity since regions with intermediate contrast were less suppressed during denoising. The latter may be beneficial for other tasks, like manual annotation of membrane-bound proteins.

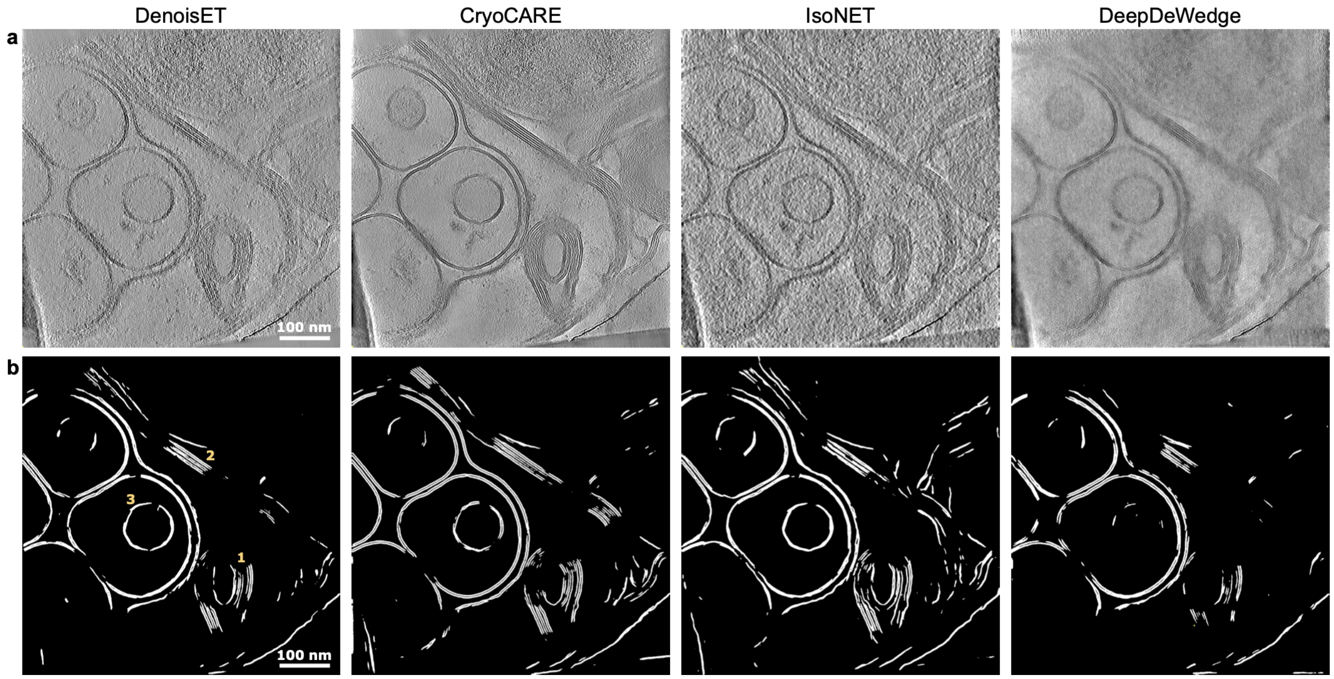

Supplemental Figure 9: **Comparison of denoising approaches on a tomogram not in the training set. a.** A slice through a tomogram denoised by the indicated software program is visualized. **b.** Membrane segmentations for the slices shown in **a** are visualized, with numbers indicating specific regions where segmentation quality diverges the most among the denoised tomograms.

References

1. Gilbert, B. R. *et al.* Generating Chromosome Geometries in a Minimal Cell From Cryo-Electron Tomograms and Chromosome Conformation Capture Maps. *Frontiers in Molecular Biosciences* **8,** 644133. ISSN: 2296-889X. https://www.ncbi.nlm.nih.gov/pmc/articles/PMC8339304/ (2025) (July 22, 2021).
2. Peck, A. *et al.* A realistic phantom dataset for benchmarking cryo-ET data annotation. *Nature Methods* **22,** 1819–1823. ISSN: 1548-7105. https://doi.org/10.1038/s41592-025-02800-5 (Sept. 2025).
3. Majtner, T. & Turoňová, B. Automated Removal of Corrupted Tilts in Cryo-Electron Tomography. *bioRxiv.* eprint: https://www.biorxiv.org/content/early/2025/03/14/2025.03.13.642992.full.pdf. https://www.biorxiv.org/content/early/2025/03/14/2025.03.13.642992 (2025).
4. Tan, X. *et al. TomoScore: A Neural Network Approach for Quality Assessment of Cellular cryo-ET* Pages: 2024.11.06.622356 Section: New Results. Nov. 8, 2024. https://www.biorxiv.org/content/10.1101/2024.11.06.622356v1 (2024).
5. Liu, H.-F. *et al.* nextPYP: a comprehensive and scalable platform for characterizing protein variability in situ using single-particle cryo-electron tomography. *Nature Methods* **20,** 1909–1919. ISSN: 1548-7105. https://doi.org/10.1038/s41592-023-02045-0 (Dec. 2023).
6. Tegunov, D. & Cramer, P. Real-time cryo-electron microscopy data preprocessing with Warp. *Nature Methods* **16.** Publisher: Nature Publishing Group, 1146–1152. ISSN: 1548-7105. https://www.nature.com/articles/s41592-019-0580-y (2024) (Nov. 2019).
7. Tegunov, D., Xue, L., Dienemann, C., Cramer, P. & Mahamid, J. Multi-particle cryo-EM refinement with M visualizes ribosome-antibiotic complex at 3.5 Å in cells. *Nature Methods* **18.** Publisher: Nature Publishing Group, 186–193. ISSN: 1548-7105. https://www.nature.com/articles/s41592-020-01054-7 (2024) (Feb. 2021).
8. Mastronarde, D. N. & Held, S. R. Automated tilt series alignment and tomographic reconstruction in IMOD. *Journal of Structural Biology* **197.** Electron Tomography, 102–113. ISSN: 1047-8477. https://www.sciencedirect.com/science/article/pii/S1047847716301526 (2017).
9. Elferich, J., Kong, L., Zottig, X. & Grigorieff, N. CTFFIND5 provides improved insight into quality, tilt and thickness of TEM samples. *eLife* **13.** Publisher: eLife Sciences Publications Limited. https://elifesciences.org/reviewed-preprints/97227 (2024) (May 1, 2024).
10. Buchholz, T.-O., Jordan, M., Pigino, G. & Jug, F. *Cryo-CARE: Content-Aware Image Restoration for Cryo-Transmission Electron Microscopy Data* Oct. 15, 2018. arXiv: 1810.05420[cs]. http://arxiv.org/abs/1810.05420 (2024).
11. Lehtinen, J. *et al. Noise2Noise: Learning Image Restoration without Clean Data* Oct. 29, 2018. arXiv: 1803.04189[cs,stat]. http://arxiv.org/abs/1803.04189 (2024).
12. Liu, Y.-T. *et al.* Isotropic reconstruction for electron tomography with deep learning. *Nature Communications* **13.** Publisher: Nature Publishing Group, 6482. ISSN: 2041-1723. https://www.nature.com/articles/s41467-022-33957-8 (2025) (Oct. 29, 2022).
13. Moran, N., Schmidt, D., Zhong, Y. & Coady, P. *Noisier2Noise: Learning to Denoise from Unpaired Noisy Data* 2019. arXiv: 1910.11908 [eess.IV]. https://arxiv.org/abs/1910.11908.
14. Wiedemann, S. & Heckel, R. A deep learning method for simultaneous denoising and missing wedge reconstruction in cryogenic electron tomography. *Nature Communications* **15.** Publisher: Nature Publishing Group, 8255. ISSN: 2041-1723. https://www.nature.com/articles/s41467-024-51438-y (2025) (Sept. 23, 2024).
15. Rangan, R. *et al.* CryoDRGN-ET: deep reconstructing generative networks for visualizing dynamic biomolecules inside cells. *Nature Methods* **21.** Publisher: Nature Publishing Group, 1537–1545. ISSN: 1548-7105. https://www.nature.com/articles/s41592-024-02340-4 (2024) (Aug. 2024).
16. Lamm, L. *et al.* MemBrain v2: an end-to-end tool for the analysis of membranes in cryo-electron tomography. *bioRxiv.* eprint: https://www.biorxiv.org/content/early/2024/01/05/2024.01.05.574336.full.pdf. https://www.biorxiv.org/content/early/2024/01/05/2024.01.05.574336 (2024).
